# Paxillin S273 Phosphorylation Regulates Adhesion Dynamics and Cell Migration through a Common Protein Complex with PAK1 and βPIX

**DOI:** 10.1038/s41598-019-47722-3

**Published:** 2019-08-07

**Authors:** Abira Rajah, Colton G. Boudreau, Alina Ilie, Tse-Luen Wee, Kaixi Tang, Aleksandar Z. Borisov, John Orlowski, Claire M. Brown

**Affiliations:** 1 Department of Physiology, McGill University, Montreal, Canada; 2 Advanced BioImaging Facility (ABIF) McGill University, 3649 Prom. Sir William Osler, Bellini Building Rm137, Montreal, QC H3G 0B1 Canada; 3 Department of Anatomy and Cell Biology, McGill University, Montreal, Canada; 4 Cell Information Systems (CIS), McGill University, Montreal, Canada; 5 Centre for Applied Mathematics in Bioscience and Medicine (CAMBAM), McGill University, Montreal, Canada

**Keywords:** Focal adhesion, Focal adhesion

## Abstract

Cell migration is an important biological phenomenon involved in many homeostatic and aberrant physiological processes. Phosphorylation of the focal adhesion adaptor protein, paxillin, on serine 273 (S273) has been implicated as a key regulator of cell migration. Here, it is shown that phosphorylation on paxillin S273 leads to highly migratory cells with small dynamic adhesions. Adhesions at protrusive edges of the cell were more dynamic than adhesions at retracting edges. Temporal image correlation microscopy revealed that these dynamic adhesions undergo rapid binding of paxillin, PAK1 and βPIX. We identified membrane proximal adhesion subdomains in protrusive regions of the cell that show rapid protein binding that is dependent on paxillin S273 phosphorylation, PAK1 kinase activity and phosphatases. These dynamic adhesion subdomains corresponded to regions of the adhesion that also show co-binding of paxillin/PAK1 and paxillin/βPIX complexes. It is likely that parts of individual adhesions are more dynamic while others are less dynamic due to their association with the actin cytoskeleton. Variable adhesion and binding dynamics are regulated via differential paxillin S273 phosphorylation across the cell and within adhesions and are required for regulated cell migration. Dysregulation through phosphomutants, PAK1-KD or βPIX mutants resulted in large stable adhesions, long protein binding times and slow cell migration. Dysregulation through phosphomimics or PAK1-CA led to small dynamic adhesions and rapid cell migration reminiscent of highly migratory cancer cells. Thus, phosphorylation of paxillin S273 is a key regulator of cell migration through recruitment of βPIX and PAK1 to sites of adhesion.

## Introduction

Cell migration is central to many processes, including cancer metastasis, wound healing and embryonic development^1–3^. The process involves a complex orchestration of more than 200 proteins that coalesce to form macromolecular structures referred to collectively as cell-matrix adhesions^4,5^. Cell-matrix adhesions serve as a physical connection between the intracellular cytoskeleton and the extracellular matrix (ECM), a link regulated through transmembrane α/β integrin heterodimers^6^. Engagement of integrin receptors with ECM proteins activates signaling cascades, resulting in recruitment of cytoplasmic proteins to the adhesion, and subsequent coupling to the actin cytoskeleton to aid in force transduction and cell movement relative to the substrate^6–9^.

In order for cells to move, adhesion structures are constantly formed, stabilized and disassembled across the cell via complex spatial and temporal cues. Adhesions are nucleated by nascent, often sub-resolution, structures at the edge of protrusive regions of the cell^10,11^. The formation of early stage nascent adhesions depends on the branched actin network in the lamellipodium, but not on myosin induced tension on the actin cytoskeleton^11,12^. As the cell travels forward past these nascent adhesions, they reside farther back from the protruding edge of the cell and, at the juncture between the lamellipodium and the lamella, they either disassemble or engage with actin filament bundles and mature into focal complexes, and later into large stable focal adhesions^11,12^.

Paxillin was one of the first adaptor molecules identified within adhesions^13^. Paxillin is a canonical adapter protein containing four LIM domains (double-zinc finger domains that regulate protein interactions) of which LIM2 and LIM3 are important for targeting paxillin to adhesions^14^. Paxillin contains five leucine- and aspartate-rich (LD) domains that act as protein binding modules^15^. It has a proline-rich region that can bind SH3 domain-containing proteins such as Src^16^. Paxillin contains many phosphorylation sites that act as substrates for kinases and phosphatases that regulate cell migration^17–19^ and adhesion dynamics^20^. In particular, phosphorylation of S273 within the LD4 domain was identified and shown to regulate cell migration^10^. Paxillin is an interesting cancer metastasis target as point mutations and upregulation of paxillin are associated with poor cancer clinical outcomes^21–24^.

In previous work, Nayal *et al*. showed that cells over-expressing a paxillin S273 phospho-mimetic (S273D) mutant form many small, highly dynamic adhesions and migrate at increased speeds relative to cells expressing wild-type (WT) paxillin^10^. In addition, the increase in adhesion dynamics and cell speed was regulated by a ternary complex formed between phospho-paxillin (pS273), GIT1 (ARF GTPase-activating protein), βPIX (PAK-Interacting eXchange factor) and PAK1 (p21-activated kinase 1). It was proposed that PAK1 phosphorylates paxillin on S273, and paxillin-pS273 subsequently binds to GIT1. GIT1 is known to form a tight complex with βPIX, which recruits PAK1 to form a quaternary complex between paxillin-pS273 and GIT/βPIX/PAK1^10,25,26^. The model suggested that PAK1 operates both upstream and downstream of paxillin S273 phosphorylation maintaining high paxillin-pS273 at sites of adhesion formation^10^. Previously, the GIT/βPIX/PAK complex has been implicated in the regulation of adhesion disassembly and in acetylcholine-induced smooth muscle contraction^27,28^.

Phosphorylation of paxillin at this site has also been implicated in other cellular processes. In rat neuronal cells, paxillin associates dynamically at adhesions in the periphery of growth cones^29^. When the neurons are injured, phosphorylation of paxillin S301, which corresponds to S273 in chicken, increased axon growth and regeneration by promoting rapid disassembly of these adhesions. Furthermore, phosphorylation of nuclear-localized paxillin at S272 in mice, has been shown to retain paxillin in the nucleus where it can participate in the regulation of gene expression and proliferation^30^.

PAK1 has also been shown to play a role in regulating adhesion dynamics, cell migration and cell protrusion in Ptk1 cells^31^. When a PAK auto-inhibitory domain protein was expressed in the Ptk1 cells, adhesion assembly and disassembly rates decreased. The adhesions remained stable for 30 minutes or more, and cell migration and protrusion were significantly reduced^31^. Furthermore, it was shown that PAK activity was negatively correlated with fibronectin density in the extracellular matrix^31^. These findings are consistent with the model proposed by Nayal *et al*.^10^.

A key role for βPIX in the regulation of adhesion dynamics and cell migration has also been shown to support lamellipodial protrusions by negatively regulating adhesion maturation and promoting rapid adhesion disassembly^32^. In keratinocytes, βPIX is localized to adhesions in the leading edge during single cell migration and is important for recruiting PAK to adhesions^33^.

To date, it is unclear how paxillin phosphorylation at S273 and the formation of this quaternary protein complex regulate cell migration at a molecular level. Here, we explored the regulation of cell migration via paxillin S273 phosphorylation using site-directed mutagenesis combined with conventional and advanced biophysical imaging tools. Paxillin phosphorylation mutants to mimic (S273D) or block (S273A) phosphorylation were generated. The role of PAK1 was explored using constitutively active (CA) or kinase dead (KD) mutants^10,34^. The role of βPIX was examined using βPIX mutants that could not bind to PAK1 (ΔSH3) or lacked guanine exchange factor (GEF) activity (LL) to abrogate the normal function of the molecular complex^10,34,35^. Pharmacological antagonists of PAK1^36^ or the serine/threonine phosphatase PP2^37^ were also used to induce hypo- or hyper-phosphorylated paxillin, respectively. Using these constructs and reagents, experiments followed cell migration tracks and adhesion assembly, stability and disassembly kinetics. The advanced biophysical techniques, image correlation microscopy (ICM) and cross-correlation microscopy (ICCM) were used to measure and spatially localize protein binding kinetics and co-binding of protein-protein complexes at sites of adhesion across the moving cell. Using this technique, we were able to perform “*in*-*situ*” biophysical and biochemical mapping of paxillin pS273 dynamics and interactions within cell matrix adhesions in live cells and identify sub-domains within adhesions that demonstrate rapid protein binding and complex formation between paxillin/PAK1 and paxillin/βPIX.

## Materials and Methods

### Cell culture and transfection

CHO-K1 cells (Sigma Aldrich, 85050302) were cultured in low-glucose DMEM containing L-glutamine, 110 mg/L sodium pyruvate and pyridoxine hydrochloride (ThermoFisher Scientific, Grand Island, NY, 11885-084). Media was supplemented with 10% vol/vol FBS (ThermoFisher Scientific, 10082-147), 1% vol/vol 100X non-essential amino acids (ThermoFisher Scientific, 11140-050), 10000 units per mL penicillin/10000 μg per mL streptomycin (ThermoFisher Scientific, 10378-016), and buffered with 25 mM HEPES (Sigma Aldrich, H0887). For stable cell lines, expression of fluorescent proteins was maintained with Geneticin-418 (ThermoFisher Scientific, 11811-031) added from 50 mg/mL stock to a concentration of 0.5 mg/mL. Stable CHO-K1 paxillin-WT-EGFP cells were a gift from the laboratory of Alan Rick Horwitz (University of Virginia, Department of Cell Biology).

For all transfections, cells were grown to approximately 75% confluence in six well plates. Lipofectamine 2000 transfection reagent (ThermoFisher Scientific, 11668-027) was used with a modified protocol. Four μg of DNA and 8 μL of Lipofectamine 2000 were diluted in phenol red free optiMEM media (ThermoFisher Scientific, 11058021) to a total volume of 150 mL in separate Eppendorf tubes. Each tube was vortexed and incubated at room temperature for 5 minutes. Subsequently, the diluted DNA and Lipofectamine were combined, mixed and incubated for 25 minutes at room temperature. The transfection mixture was combined with 1.7 mL of optiMEM and added to one of the wells in a 6 well plate. The cells were incubated with DNA and Lipofectamine 2000 for 5 hours, and then the solution was replaced with full culture media.

Stable cell lines were generated for expression of the paxillin mutant (S273A, S273D) constructs. This was accomplished by transfecting CHO-K1 cells and maintaining them in media supplemented with Geneticin 418, as described above for stable cell lines, maintained for two weeks, and using fluorescence activated cell sorting (FACS) to sort cells expressing the constructs at levels close to endogenous.

### Plasmids

All experiments were performed on CHO-K1 cells with paxillin constructs fused to EGFP and PAK1 or βPIX constructs fused to mCherry. To simplify the text, the EGFP and mCherry indications are not included in the nomenclature of the constructs. The fluorescent proteins are mentioned in this section and in the figure captions. Paxillin-S273A-EGFP, Paxillin-S273D-EGFP, paxillin-WT-EGFP-mCherry, βPIX-WT-HA, βPIX-LL-HA, βPIX-ΔSH3, PAK1-WT, PAK1-CA and PAK1-KD constructs were gifts from the laboratory of Alan Rick Horwitz^10^. All paxillin constructs came from chicken while βPIX and PAK1 constructs were from mouse. βPIX and PAK1 constructs were subcloned from their parent vectors and inserted in a pAc-mCherry-C1 vector. PCR primers were designed and obtained (Integrated DNA Technologies, Coralville, Iowa) for βPIX containing Xma1 and Kpn1 restriction sites (5′-CGG GGT ACC GCC GCG ATG ACT GAT AAC A-3′ and 5′-TCC CCC CGG GCT ATA GAT TGG TCT CAT CC-3′) and PAK1 (5′-CGG GGT ACC GCC GCG ATG TCA AAT AAC G-3′ and 5′-TCC CCC CGG GTC AGT GAT TGT TCT TGG TT-3′). The PCR product was purified using 1% agarose gel electrophoresis and digested along with the empty mCherry with XmaI (New England Biolabs, Ipswich, Massachusetts, CAT# R0180S) and KpnI-HF (New England Biolabs, CAT# R3142S). The digested products and vector were purified using a 1% agarose gel electrophoresis, and the mCherry vector was subjected to Calf Intestinal Phosphatase (New England Biolabs, M0290S) treatment. The insert was ligated into the mCherry vector using T4 DNA ligase (New England Biolabs, M0202L), transformed, cultured, mini-prepped (Qiagen, 27104) and sequenced (Genome Quebec).

### Antibodies and reagents

The following primary antibodies were used for immunoprecipitation and western blot experiments: polyclonal anti-GFP (ThermoFisher Scientific, A6455), unconjugated rabbit IgG isotype control antibody (Southern BioTech, 0111-01), paxillin monoclonal (Sigma Aldrich, P1093), monoclonal anti-mCherry (Abcam, Cambridge, UK, ab125096) and monoclonal anti-β-Tubulin (Sigma Aldrich, T0198). Goat anti-mouse horseradish peroxidase- (HRP) conjugated secondary antibodies were purchased from Jackson ImmunoResearch Laboratories (West Grove, PA, 115-036-062). Unless specified, all other reagents for immunoblotting were obtained from BioShop Canada (Burlington, ON, Canada). IPA-3 was obtained from Cayman Chemicals (42521-82-4) and dissolved in DMSO. Okadaic acid sodium salt from *Prorocentrum concavum* (Sigma Aldrich, O7760) was dissolved in ddH_2_O. TetraSpeck 0.2 μm microspheres were obtained from ThermoFisher Scientific (T7280). Phalloidin conjugated to Alexa-Fluor® 594 was obtained from Life Technologies (A12381).

### Immunoprecipitation and western blot experiments

CHO-K1 cells stably expressing paxillin-EGFP were cultured to 75% confluency in 10 cm dishes and transfected with 4 μg of PAK1-mCherry or βPIX-mCherry plasmids using Lipofectamine 2000. Twenty-four hours post-transfection, cells were washed twice with ice-cold PBS and scraped with 500 μl of lysis buffer containing PBS, 1% Nonidet-P40, 0.25% sodium deoxycholate, 1 mM EDTA and mini protease inhibitor cocktail (Roche). The lysed cells were rocked for 30 minutes, centrifuged for 20 minutes at 13,200 × *g* at 4 °C and the supernatants were collected. An aliquot of each lysate was kept for total lysate analyses. Protein supernatant were pre-cleared on protein G-Sepharose beads (GE Healthcare) for 2 hours at 4 °C. After centrifugation to remove the beads, the pre-cleared supernatants were incubated with 3 μL of anti-GFP polyclonal antibody overnight at 4 °C. The next day, cells were incubated with a 50% slurry of Protein G-Sepharose for 3 hours at 4 °C and then washed five times with lysis buffer. The immunoprecipitates and whole-cell lysates were subjected to sodium dodecyl sulfate polyacrylamide gel electrophoresis (SDS-PAGE) on 10% gels and transferred overnight onto polyvinylidene fluoride (PVDF) membranes (Millipore, Ontario, Canada). The membranes were blocked using 5% skim milk/PBS for one hour to prevent non-specific binding, and incubated for 1 hour with the indicated primary and secondary antibodies in 5% skim milk/0.1% Tween 20/PBS at the following concentrations: monoclonal paxillin 1:4000, anti-mCherry 1:2500, β-tubulin 1:10,000 and HRP-conjugated anti-mouse at 1:5000. Membranes were washed several times in 0.1% Tween/PBS before and after each antibody incubation. Western Lightning Plus ECL reagent (Perkin Elmer, Inc., Waltham, MA) was used to visualize the immunoblot bands. The intensity of the bands was quantified by densitometry of X-ray films exposed in the linear range and analyzed using ImageJ (NIH).

### Live cell imaging preparation

For all live cell experiments, 35 mm glass bottom dishes (World Precision Instruments, Sarasota, FL, FD35) were coated with 2 μg/mL fibronectin (Sigma Aldrich, F0895) diluted with warm PBS for 1 hour at 37 °C under 5% CO_2_. Dishes were then washed twice with warm PBS and 25,000 cells were plated on the dish in tissue culture media.

### Cell tracking assays

Paxillin-EGFP WT, S273A, and S273D stable cell lines were plated on fibronectin coated µ-Slide 8 Well imaging slides (ibidi, Cat#80826). Cells were incubated for 2–3 hours and then placed in a microscope stage top environmental control chamber (Live Cell Instrument, Seoul, Korea), maintained at 37 °C under a 5% CO_2_ humidified environment with a flow rate of 50 mL/min. The chamber was placed on the stage of an inverted microscope (AxioObserver, Carl Zeiss) with an Axiocam 506 monochrome camera (Zeiss) and 20x/0.5 NA objective lens (Carl Zeiss). Phase contrast transmitted light imaging with exposure times of 150–300 ms were used to acquire all images. A multi-dimension acquisition using phase contrast mode was programmed using AxioVision 4.8.2 software, where five sites per well were chosen and imaged every 10 minutes for a total of 18 hours.

For multiple wavelength tracking experiments, PAK1- or βPIX-mCherry fusions were transfected into CHO-K1 paxillin-EGFP-WT stable cells. These cells were allowed to recover for 24 hours and then plated on 96 well plates (Corning, 3882), incubated for 2–3 hours and imaged using a high content screening device (ImageXpress XL System, Molecular Devices, Sunnyvale, CA) under identical conditions (37 °C and 5% CO_2_) using the live cell plate gasket of the unit. A 20x/0.45 NA objective lens (Nikon), Chroma EGFP (49002) and Texas Red (49008) filter cubes with 6% excitation power and an exposure time of 150 ms was used to acquire images in four sites per well in both the EGFP and mCherry channels every 10 minutes for 18 hours.

Cell tracking data was obtained by loading the sequential images as a stack in MetaXpress 5.0 and manual cell tracking was carried out using the “*track points*” function. A minimum of 45 cells were tracked for each experiment for a minimum total track length of 6 hours. The data was logged into Excel (Microsoft) and average cell speed in hours was calculated by dividing the distance moved between frames by 1/6 of an hour. The average speed of each cell was calculated by averaging the instantaneous speed between each frame tracked over the 6-hour period.

### Total internal reflectance fluorescence (TIRF) microscopy

To measure adhesion dynamics, single-wavelength temporal image correlation microscopy (tICM) and dual-wavelength temporal image cross correlation microscopy (tICCM) assays were carried out using a TIRF microscope platform. Cells on 35 mm cover glass bottom dishes were placed in a stage top environmental control chamber (Live Cell Instrument, CU-501) at 37 °C under a 5% CO_2_ environment with a flow rate of 50 mL/min. TIRF was achieved using a Spectral Diskovery unit (Spectral Applied Research, Richmond Hill, ON) attached to an inverted Leica DMI6000B microscope (Leica Microsystems, Wetzler, Germany) with a Leica Plan ApoChromat 63x/1. 47 NA TIRF oil immersion objective lens. The platform incorporated a 488 nm diode laser, 561 nm diode-pumped solid-state laser (Spectral Applied Research) and was equipped with two ImagEMX2 Digital EM-CCD Cameras (Hamamatsu, Hamamatsu City, Japan). An EGFP filter cube (ET 525/50 nm), mCherry filter cube (ET 620/60 nm), and EGFP/mCherry dual cube allow for single wavelength or dual-wavelength simultaneous pixel-by- pixel image acquisitions. A 100 Watt X-Cite 120 LED (370–700 nm) source was also integrated to allow visualization of fluorescence proteins by eye. The platform was integrated with MetaMorph 7.1 image acquisition software (Molecular Devices Inc.) and custom designed TIRF controls (Quorum Technologies Inc., Guelph, ON).

### TIRF adhesion dynamics, stability and size assays

For paxillin-EGFP WT and mutant experiments, cells were located using the eyepieces, the TIRF prism was put in place and the penetration depth was set to ~80 nm using the software interface. A multi-dimensional acquisition was set up to acquire an image every 30 seconds for a total of 30 minutes. The 488 nm laser was set to 10% with 10% laser pulsing using the Quorum Flicker device for a total laser power of ~1%. Exposure time was set to 150 ms and the camera read speed was 22 MHz and the EM gain was set to 180 on the 0–255 gain scale.

For assays monitoring the effect of PAK1 and βPIX mutants on paxillin dynamics, each of the mCherry constructs were transiently transfected in CHO-K1 paxillin-EGFP WT stable cells. Cells were located by eye on the TIRF platform to select for cells expressing both constructs. These cells were then subjected to a multi-dimensional image acquisition of only the paxillin EGFP fluorescence, as described above.

To measure adhesion assembly and disassembly rates, the image stacks were opened in MetaXpress 5.0. The background intensity was subtracted from each image frame using the intensity of an ROI in an area of the image with no cells. The MetaXpress statistical background correction module was used for this and a custom journal was created to loop the image processing for each plane of the image stack. The corrected file was saved as a TIF stack and opened in Imaris 7.5.2 (Andor Technology, Belfast, Ireland) as a time series. The surfaces function was employed to identify and measure adhesion intensities.

The average intensity versus time series for each adhesion was plotted using a custom macro program in Microsoft Excel. Regions of the plot where assembly (increasing intensity versus time) and disassembly (decreasing intensity versus time) were cropped and a second custom macro program was used to select the data and plot ln(intensity/minimum intensity value) versus time for assembly and ln(maximum intensity value/intensity) versus time for disassembly^38^. The slope and R^2^ correlation value was recorded for each natural semi-logarithmic plot. The slope corresponded to the rate of assembly or disassembly. Adhesion dynamic data was rejected for assembly or disassembly plots with an R^2^ < 0.7. A minimum of 20 adhesions undergoing assembly or 20 adhesions undergoing disassembly were obtained for each cell. Each experiment included 4 to 5 cells. Adhesion stability times were manually recorded by analyzing raw intensity versus time series and counting the number of frames where the intensity was not changing. Average adhesion size data was obtained by selecting one image plane from the time series, measuring the area of each adhesion using Imaris and taking the average adhesion size for the analyzed region.

### Temporal image correlation (tICM) assays

The tICM technique is also known as image fluorescence correlation spectroscopy (image FCS) and was recently reviewed^39^. We first demonstrated camera-based FCS or tICM (TIRF-tICS) on paxillin in adhesions and showed that paxillin in assembling areas of adhesions had faster dynamics while disassembling areas showed slower dynamics^40^. A detailed protocol for imaging FCS/tICM and fluorescence cross-correlation spectroscopy (FCCS) which is analogous to tICCM was also recently published^41^. Briefly, the intensity at an individual pixel location through an image time series will fluctuate. The fluctuations in intensity are related to movement of fluorescent signals in and out of the focal volume associated with that pixel. In our case with TIRF microscopy, fluctuations in intensity are due to fluorescent protein fusions moving into and out of the TIRF illumination field. For our purposes, this corresponds to binding of proteins to adhesions. Protein movement in the cytosol is too fast to be measured under the conditions used here. A statistical analysis of the intensity fluctuations at each pixel will generate an autocorrelation function (ACF) and from a fit to that ACF the dynamics of the binding can be measured^41^. This can be done for every pixel across the image.

Image acquisitions were conducted using TIRF microscopy, wherein cells were selected and imaged with a penetration depth of ~80 nm. To deduce regions of cell protrusion and retraction, a multi-dimensional acquisition time series of cell movement was recorded prior to the tICM image acquisition. EGFP images were collected every minute for 7 minutes with the exposure time set to 75 ms and the 488 nm laser power set to 7%. Regions of protrusion or retraction were identified from the 7-image time series. For the tICM assay, each cell was imaged using the stream acquisition option in the MetaMorph software. It was set to collect 1000 frames with a 30 ms interval. Due to the camera read time, there is a 1 ms delay every 10 frames with a 30 ms frame rate. This corresponds to a 1 ms delay every 300 ms or a 0.33% error in the time measurements. The 488 nm laser line was set at 10% power for EGFP constructs. The pulsing feature of the Flicker was disabled to avoid potential interference of laser pulsing with intensity fluctuations due to protein movement. The 561 nm laser was used at 12% power for mCherry constructs. The camera was set to read at 22 MHz, the EM gain was set to 180 out of 255 and exposure time corresponded with the frame acquisition time of 30 ms.

The image background was corrected as above and the corrected stacks were saved as TIF stacks and subsequently opened in the 64-bit version of the SimFCS software (Globals Software, Laboratory for Fluorescence Dynamics, Irvine, CA). Briefly, image stacks were opened in the SimFCS software. The average cytosolic intensity was noted from MetaMorph and the image stack was thresholded above that intensity value on the SimFCS software until only adhesions were visible and no cytosolic or background areas were present. The first 10–20 images in the time series were deleted because of excessive bleaching during that period. A moving average of 10 frames was applied to the analysis to ensure that any slow physical movement of the cell or the adhesions themselves between image frames would not introduce artifacts in the protein dynamic data. To obtain the exponential times related to the protein binding kinetics the phasor analysis was used. This analysis does not require any data fitting but pulls the binding kinetics directly from the raw data^42^. A binding and unbinding model was selected, as it is measuring protein binding to adhesions rather than protein diffusion. An exponential time histogram was generated for all of the pixels within adhesions. This histogram could be colour coded by rate and mapped back onto the original image data (Supplemental Fig. S3).

### Temporal image cross-correlation microscopy (tICCM) assay

Similar to tICM, tICCM is a statistical analysis of the intensity fluctuations of two different fluorescent signals that will only lead to a cross correlation function (CCF) if the two signals are co-incident^41^. In our case, this is when the EGFP and the mCherry protein fusions are binding and unbinding adhesions together. The technique does not prove direct binding between the proteins but does show co-dynamics within the same protein complex. CHO-K1 cells stably expressing paxillin-EGFP were transfected with mCherry labeled constructs of interest. The cells were imaged on the TIRF platform with a GFP/mCherry long pass dual dichroic (Chroma Technologies Inc., T565lpxr) and emission filters for EGFP (ET525/50 M) and mCherry (ET620/60 M) before each EMCCD camera. It was essential for accurate cross-correlation analysis that the cameras were perfectly aligned immediately prior to all experiments. To ensure pixel-to-pixel overlap a sample of four colour TetraSpeck microspheres was suspended in water. Stream acquisition mode and the MetaMorph driver for true simultaneous dual camera exposure and read out was used. Two simultaneous image stacks consisting of 275 frames each were obtained with a frame rate of 30 ms. The exposure time corresponded with the frame acquisition rate of 30 ms and the cameras were set to read out at 22 MHz with the EM gain for the EGFP channel set to 185 and the EM gain for the mCherry channel set to 220. The laser power was set to 7% for both the 488 nm and 561 nm lasers. Prior to each dual wavelength stream acquisition, a paxillin-EGFP image time series was obtained in an identical manner to the tICM multi-dimensional acquisitions in order to determine regions of protrusion and retraction.

Each image time series was loaded in the SimFCS software and processed for tICM for the EGFP and the mCherry labelled proteins as outlined above. To generate cross-correlation maps, The CCF was displayed in the lower right corner of the software window, and the phasor file showing dynamics and pixel locations where cross-correlation was measured was logged to Excel. More details on the analysis are found in the Supplemental Materials.

### Confocal images and protein quantification at adhesions

Cells co-expressing paxillin-EGFP and transiently transfected βPIX-mCherry or PAK1-mCherry were plated on fibronectin-coated 35 mm dishes (World Precision instruments, FD-35). They were visualized on a Zeiss LSM710 microscope with a Plan-ApoChromat 63x/1.4 NA oil immersion objective lens. Sequential imaging was conducted using 1% laser power from the 488 nm line from a 25 mW Argon-Ion laser to excite EGFP and a 20 mW diode-pumped solid state (DPSS) laser at 561 nm to excite mCherry. Imaris 8.3 was used to perform quantitative intensity analysis of adhesion and non-adhesion areas. The “surface” function was used to generate a surface that uses the automatic thresholding function to detect the adhesions in the images. A reference non-adhesion surface was drawn beside a selected adhesion at a similar size. The intensity measurement of the adhesion and near adhesion regions were extracted to measure percentage enrichment of the proteins in adhesions as follows: % enrichment = (F_adhesion_ − F_non-adhesion_)/F_non-adhesion_. The histogram function in Microsoft Excel was used to generate distribution curves for each condition.

### Fluorescence recovery after photobleaching (FRAP) experiments

FRAP experiments were conducted with a Zeiss LSM710 with a Plan-ApoChromat 63x/1.4 NA oil immersion objective lens. Transfected cells were plated on fibronectin-coated 35 mm dishes and were maintained in a stage-top humidified environmental chamber during acquisition. Cells expressing detectably low levels of the transfected fluorescent proteins were chosen for the bleaching acquisitions in order to minimize protein overexpression artifacts. Since PAK1-mCherry and βPIX-mCherry do not readily localize at focal adhesions in a visible way, adhesions were first outlined on the paxillin-EGFP channel using the region tool on the ZEN software. After switching to the mCherry channel, the bleach settings were set up to acquire, with 0.39 μs pixel dwell times, 10 pre-bleach images with 1% laser power from the 561 nm laser, followed by 3 bleach iterations at 100% intensity and, lastly, post-bleach recovery at 1% laser power. At a zoom setting of 2.0, the scan area was set at 1024 × 50 pixels and the acquisition time took 47.5 ms with a 1.7 ms delay between each frame. The FRAP intensity data were exported from ZEN and logged onto Excel. The raw intensity values from the recovery data was background subtracted and divided by the intensity of an unbleached acquisition region to correct for any basal levels of bleaching caused by the acquisition process itself or focal drift. The average normalized recovery data of multiple bleached adhesion and non-adhesion areas were plotted to generate FRAP recovery curves. Origin 9.3 software (OriginLab Corporation, MA) was used to approximate the recovery curve to a simple exponential curve: F(t) = F_mobile_ • (1–e^τ•t^). The immobile fraction was calculated by the following formula: F_immobile_ = 1 − F_mobile_. To study FRAP in fixed cells, the transfected cells were fixed with 4% para-formaldehyde (PFA) for 15 minutes and left in PBS and then imaged the following day. Fixed cell data were used to estimate the first post-bleach data point due to rapid fluorescence recovery during the image acquisition time.

## Results

### Cell migration is regulated by paxillin S273 phosphorylation and depends on active PAK1, βPIX and phosphatases

**Figure 1:**
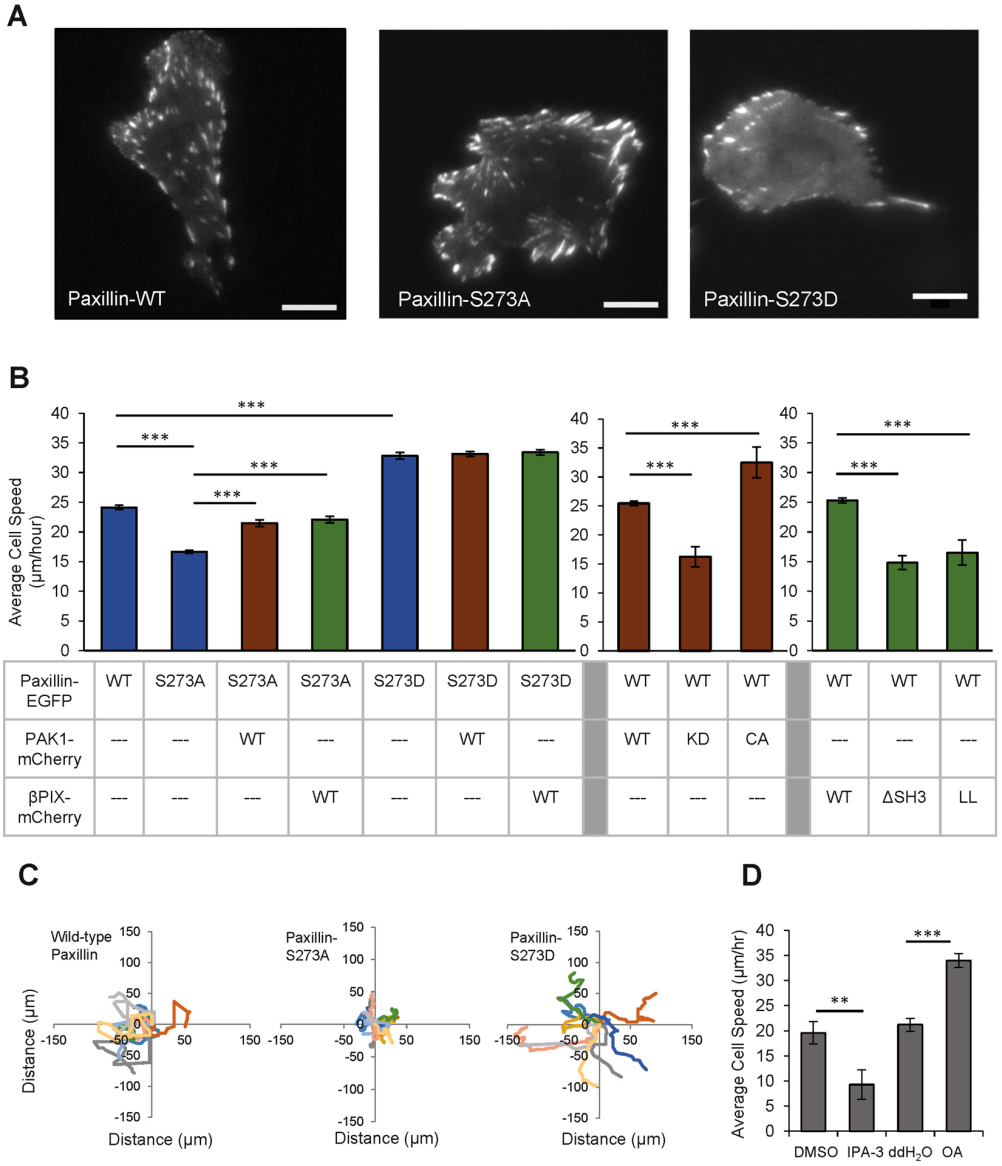
Paxillin S273 phosphorylation combined with PAK1 kinase activity, βPIX GEF activity, βPIX binding to PAK1 and phosphatase activity regulate CHO-K1 cell migration. (**A**) Representative images of CHO-K1 cells expressing paxillin-WT-EGFP (left), -S273A (middle), and -S273D (right). Scale bars for images are 5 μm. (**B**) Cell migration speed expressing paxillin-WT, –S273A, -S273D with expression of PAK1-WT, -KD,-CA or βPIX-WT, -ΔSH3, -LL. (**C**) Rose plots of 8 representative cell migration paths of cells expressing paxillin-WT, -S273D, -S273A fused with EGFP. (**D**) Migration speed of paxillin-WT-EGFP cells treated with 10 μM IPA-3 (PAK1 kinase inhibitor) or 50 nM okadaic acid (PP2a phosphatase inhibitor). Error bars represent SEM. Two stars (**) corresponds to p < 0.001, three stars ***p < 0.0001. n = 90 cells, 30 cells from three independent experiments.

To explore the effect of paxillin phosphorylation at S273 on single cell migration, experiments were performed on cells stably expressing paxillin-WT, –S273A or –S273D mutants (Fig. 1A). Cells were plated at a low density to follow single cell tracks for six hours. When compared to cells expressing paxillin-WT, the expression of the non-phosphorylatable paxillin-S273A mutant hindered cell migration speed by 28% while the phospho-mimetic paxillin-S273D mutant induced cells to move 27% faster (Fig. 1B,C). Cell migration speeds with paxillin-S273A expression were recovered by overexpression of PAK1 or βPIX (Fig. 1B). This is likely due to enhanced activation of endogenous paxillin by the overexpressed proteins. Overexpression of PAK1 or βPIX, however, did not affect migration rates in cells expressing paxillin-S273D, presumably because they were already maximally stimulated (Fig. 1B). To determine if the migration phenotype associated with paxillin phosphorylation at S273 was dependent on PAK1 kinase activity, experiments were performed with cells expressing paxillin-WT and either WT, constitutively active (CA) or kinase dead (KD) PAK1. PAK’s role in paxillin phosphorylation was clear as cells overexpressing PAK1-CA showed a 38% increase in cell speed, whereas cells overexpressing PAK1-KD had a 32% decrease in cell speed (Fig. 1B, Supplemental Fig. S1A). Pharmacological inhibition of PAK1 kinase activity with IPA-3 reduced cell speed by 53% (Fig. 1D), whereas blocking endogenous paxillin dephosphorylation with okadaic acid^37^, a protein phosphatase-2 (PP2) inhibitor, increased cell speed by 60% compared to control conditions (Fig. 1D). Collectively, these data support a critical role for PAK1-mediated phosphorylation of paxillin in regulating cell migration velocity.

To investigate the role of βPIX in regulating cell migration, βPIX mutants that are unable to bind PAK1 (ΔSH3) or lack guanine exchange factor (GEF) activity (LL) were co-expressed with paxillin-WT. Cells expressing βPIX-ΔSH3 or –LL exhibited reductions in cell migration speed (28% and 36%, respectively) (Fig. 1B, Supplemental Fig. S1B). These experiments indicate that βPIX binding to PAK1 and its GEF activity are required to regulate cell migration speeds.

Past studies have shown that paxillin S273 phosphorylation regulates cell migration^10^ and that PAK inhibition leads to decreased cell migration^31^. Here we confirm these previous results but also show that overexpression of PAK1 or βPIX can rescue the paxillin-S273A phenotype, that βPIX GEF activity and binding to PAK1, and phosphatases are important for the regulation of cell migration by paxillin S273 phosphorylation.

### Phosphorylation of paxillin S273 induces small, dynamic adhesions that are regulated by active PAK1, βPIX and phosphatases

**Figure 2:**
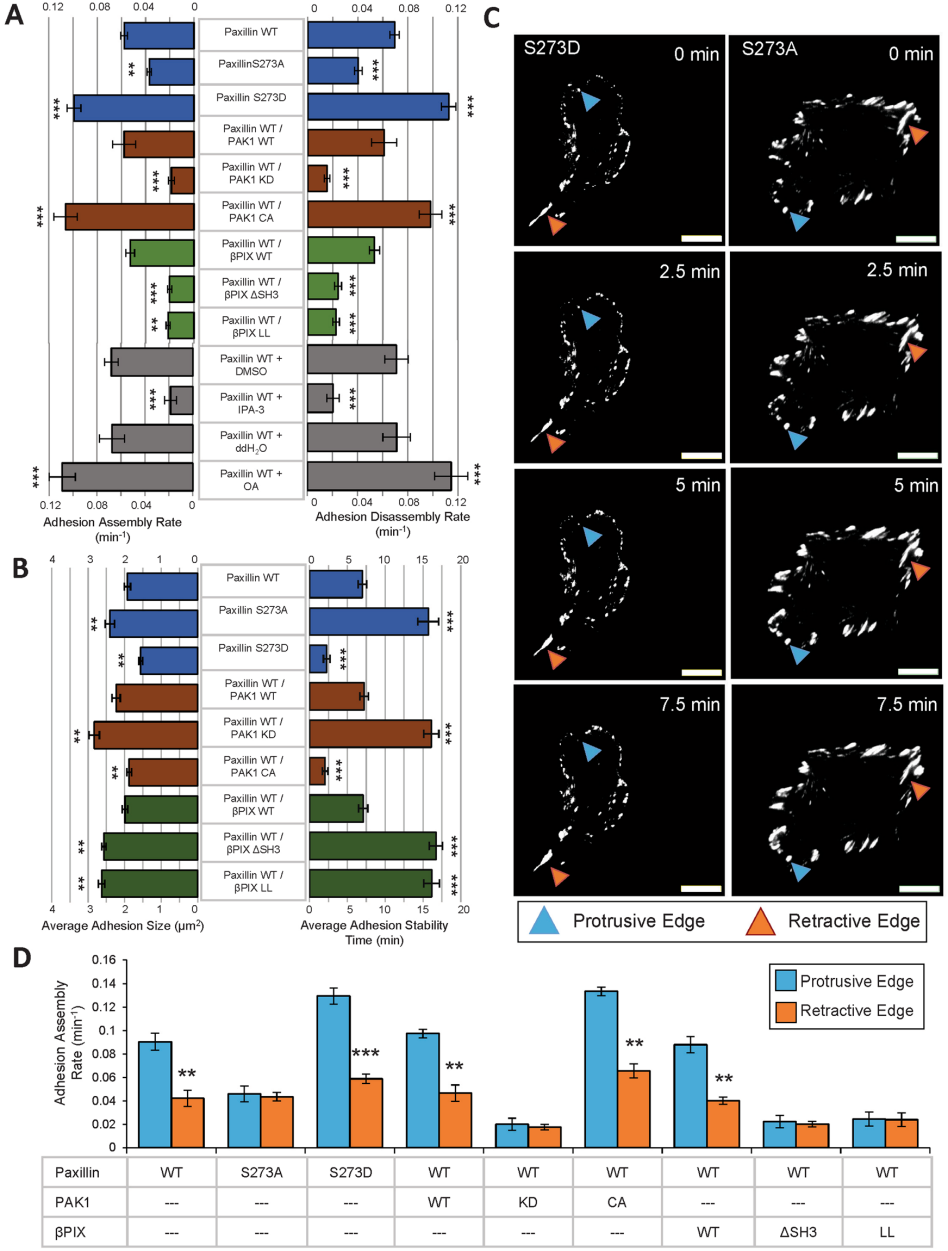
Paxillin S273 phosphorylation, PAK1 kinase activity, βPIX GEF activity, βPIX binding to PAK1 and phosphatase activity regulate adhesion dynamics. (**A**) Adhesion assembly rates (left) and disassembly rates (right) for cells expressing paxillin-WT, -S273A, -S273D (blue), co-expression of paxillin-WT and PAK1-WT, -KD, -CA (red), co-expression of paxillin-WT and βPIX-WT, -ΔSH3, -LL variants (green), and cells with paxillin-WT treated with 10 μM IPA-3 or 50 nM okadaic acid (grey). (**B**) Average adhesion size (left) and average adhesion stability time (right) for cells expressing paxillin variants (blue), co-expressing paxillin and PAK1 variants (red), and co-expressing paxillin-WT and βPIX variants (green). (**C**) Time series of cells expressing paxillin-S273D (left panel) and -S273A (right panel). Cyan arrowhead indicates protrusive edges, orange arrowhead indicates retractive edges. Scale bars are 5 μm. (**D**) Adhesion assembly rates at protrusive (cyan bars) or retractive (orange bars) edges of the cell. Error bars represent SEM. Two stars (**) corresponds to p < 0.001, three stars ***p < 0.0001. n = 45–60 adhesions. n = 15–20 adhesions, N = 3–5 cells per experiment and 3 independent experiments.

The dynamic process of cell-matrix adhesion assembly and disassembly was characterized for paxillin using total internal reflectance fluorescence (TIRF) microscopy. Adhesion assembly and disassembly rates, stability and size were measured. Nascent adhesion dynamics could not be measured on the timescale of these experiments due to their rapid lifetime of less than one minute. Instead, the analyses focused on intermediate sized focal complexes, with the exception of the S273A mutant which formed mostly larger focal adhesions. Expression of non-phosphorylatable paxillin-S273A resulted in large stable adhesions with a 75% decrease in adhesion assembly rates and 68% decrease in adhesion disassembly rates (Fig. 1A, 2A,B) when compared to paxillin-WT. By contrast, expression of phospho-mimetic paxillin-S273D induced small, dynamic adhesions with increased assembly and disassembly rates by 62% and 72%, respectively (Fig. 2A,B). These results are consistent with previous work^10^ with the inclusion of additional paxillin/PAK1/βPIX combinations and additional metrics such as adhesion size and stability.

To determine if the kinase activity of PAK1 was essential for the observed phenotype, paxillin-WT expressing cells were transfected with PAK1-WT, -CA or -KD. Adhesion dynamics were measured from the intensity of the EGFP signal from paxillin-WT. Overexpression of PAK1-CA mimicked cells expressing paxillin-S273D, exhibiting small adhesions with fast assembly and disassembly rates and short stability times as compared to cells expressing PAK1-WT (Fig. 2A,B). Conversely, overexpression of PAK1-KD mimicked cells expressing paxillin-S273A and displayed large and stable adhesions with slow assembly and disassembly rates (Fig. 2A,B). The reduced adhesion assembly and disassembly rates are consistent with rates measured in PtK1 cells expressing a PAK1 autoinhibitory domain^31^.

The role of βPIX binding to PAK1 and its GEF activity on paxillin-WT adhesion size and dynamics was explored by co-expression of βPIX-ΔSH3 and -LL mutants, respectively. Adhesion characteristics were measured only in those adhesions containing paxillin-WT. Impaired βPIX-PAK1 binding (ΔSH3) or βPIX GEF activity (LL) significantly decreased adhesion dynamics and resulted in the formation of large stable adhesions (Fig. 2A,B).

Pharmacological inhibition of PAK1 or PP2 phosphatase with IPA-3 and okadaic acid, respectively, resulted in phenotypes similar to those obtained with the corresponding hypo-phosphorylatable (S273A) and phospho-mimetic (S273D) paxillin mutants (Fig. 2A), thereby implicating the involvement of endogenous PAK1 and PP2 and minimizing concerns of artifacts based on exogenous gene overexpression. Collectively, these data support a crucial regulatory role for phosphorylation of endogenous paxillin at S273 in adhesion dynamics.

### Paxillin pS273 plays a role in differential adhesion dynamics across the cell

Protrusive (Fig. 2C, blue arrowheads) and retracting edges (Fig. 2C, red arrowheads) of cells were identified from visualization of time-lapse image series. Cells expressing paxillin-WT displayed smaller, more dynamic adhesions at protrusive edges, compared to retracting edges, an effect that was accentuated by expression of paxillin-S273D (Fig. 2C,D). Conversely, cells expressing paxillin-S273A had protrusive or retractive regions that were not as dynamic and tended to have large stable adhesions across the entire cell (Fig. 2C,D). The difference in adhesion dynamics in protrusive versus retractive regions of the cell was dependent on PAK1 kinase activity (as revealed by PAK1-WT and –CA overexpression), but was not noticeably affected by PAK1-KD overexpression presumably because all of the adhesions were more stable (Fig. 2D). Differential adhesion dynamics across the cell were also observed with βPIX-WT overexpression but were negated by overexpression of the βPIX-ΔSH3 or -LL mutants (Fig. 2D). Adhesion disassembly rates showed a very similar trend compared to assembly rates for all conditions tested (Supplemental Fig. S2).

### Paxillin phosphorylation at S273 promotes differential binding rates across the cell and asymmetric binding kinetics across individual adhesions

To measure the binding dynamics of paxillin at individual adhesions, we used temporal image correlation microscopy (tICM). This technique can measure protein binding kinetics within individual pixel locations from the fluorescence intensity fluctuations between images in a time series^40,41^. An autocorrelation function (ACF) can be calculated from a statistical analysis of the intensity fluctuations within each pixel location over time. The ACF can then be fit to determine correlation decay rates and measure binding kinetics in individual image pixels^40^. tICM was applied to TIRF microscopy image time series data to quantify and spatially map the millisecond binding dynamics of paxillin-EGFP constructs at individual sites of adhesion across the cell. A typical experiment was one minute long, so entire adhesion complexes did not move significantly. The binding dynamics were expressed in terms of the binding rates in seconds^−1^ as determined from the tICM autocorrelation function (ACF) analysis.

**Figure 3:**
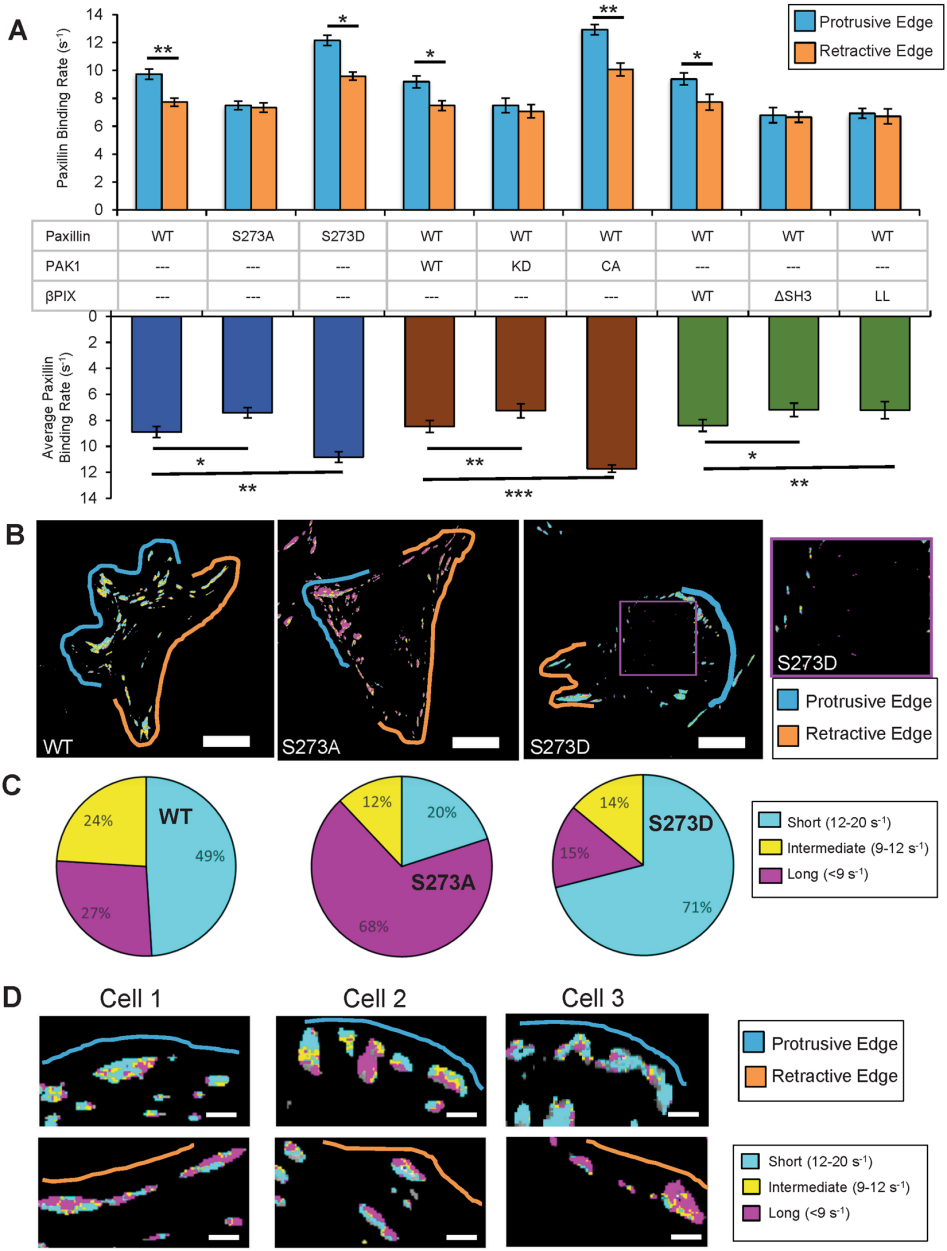
Phosphorylation of paxillin S273, PAK1 kinase activity, βPIX GEF activity and βPIX binding to PAK1 regulate paxillin binding at sites of adhesion. (**A**) Average paxillin binding rates across the cell (lower bars) for paxillin variants (dark blue bars), PAK1 variants (red bars) and βPIX variants (green bars). Paxillin binding rates at protrusive (cyan bars) and retractive (orange bars) edges of the cell (upper bars). Error bars represent SEM. One Star (*) corresponds to p < 0.01, two stars **p < 0.001, three stars ***p < 0.0001. N = 30 cells from three independent experiments. n = 10–20 adhesions in each cell identified by thresholding and analyzed. (**B**) Spatial maps of paxillin binding dynamics across cells expressing paxillin-WT (left), paxillin-S273A (middle), and paxillin-S273D (right). The protrusive edges are indicated with a cyan line, retractive edges are indicated with an orange line. Regions of short binding times (12–20 s^−1^) shown in cyan, intermediate binding times (9–12 s^−1^) in yellow and slow binding times (<9 s^−1^) in magenta. Zoomed image insert represents long-binding time small adhesions in the paxillin-S273D expressing cells. Scale bars are 5 μm. (**C**) Percentage of pixels across multiple cells with binding dynamics in defined binding time bins as in B. (**D**) Representative spatial dynamic maps of paxillin binding to adhesions in three different cells expressing paxillin-WT at protrusive (cyan lines) and retractive (orange lines) edges. Scale bars are 2 μm.

Compared to paxillin-WT expressing cells, average paxillin binding rates across the cell were faster for paxillin-S273D and slower for paxillin-S273A (Fig. 3A, bottom panel). Most interestingly, paxillin binding dynamics were faster at protrusive edges of the cell as compared to retractive edges and this differential binding rate was dependent on paxillin S273 phosphorylation (Fig. 3A, upper panel). These binding rates align well with rates measured by FRAP for the “fast” fraction within adhesions made by Wolfenson *et al*.^43^ using a focused laser beam point capable of measuring rapid protein dynamics. Wolfenson *et al*.^43^ describe this population as paxillin undergoing attenuated diffusion due to rapid binding and unbinding at the adhesion site.

Paxillin-WT binding dynamics at adhesions were measured in cells that co-expressed WT or mutants of PAK1 and βPIX. Over-expression of PAK1-CA led to an increase in the paxillin binding rate, similar to the paxillin-S273D mutant, whereas expressing PAK1-KD gave a phenotype similar to the paxillin-S273A mutant (Fig. 3A). Overexpression of βPIX proteins demonstrated that the regulation of paxillin binding at adhesions and the establishment of differential binding rates across the cell were also dependent on βPIX binding to PAK1 (βPIX-ΔSH3) and its GEF activity (βPIX-LL) (Fig. 3A). Overexpression of PAK1-WT or βPIX-WT gave dynamic binding rates identical to those obtained with cells expressing only endogenous proteins (Fig. 3A).

To understand further the extensive quantitative data generated by the tICM analysis, the average binding rates for each pixel in the image were divided into three sub-categories and colour-coded accordingly. Rates corresponding to short (12–20 s^−1^), intermediate (9–12 s^−1^) and long (<9 s^−1^) dwell times for protein binding at the adhesion were colour-coded cyan, yellow and magenta, respectively (Fig. 3B). Cells expressing paxillin-S273A showed mostly long dwell times (magenta), whereas those expressing paxillin-S273D exhibited mostly short dwell times (cyan). On the other hand, cells expressing paxillin-WT showed a mix of all three populations of binding (Fig. 3B). As seen in Fig. 3B, differences in binding rates were clearly visible in the images of paxillin-WT versus the S273A or S273D mutants. However, based on a histogram display of the binding data it is difficult to appreciate the differences between the three proteins (Supplemental Fig. S3A,B). A normalization of the histogram data accentuates the differences, but they are not as apparent as the visual cellular maps (Supplemental Fig. S3C). Thus, the data shown here demonstrates that it is important to look at the spatial distribution of these protein-binding populations across the cell. For example, upon closer inspection, it is clear that the population of adhesions with long binding dwell times in the paxillin-S273D expressing cells are mostly in the center of the cell (Fig. 3B, zoomed inset). A statistical analysis across multiple cells of the percentage of pixels with short, intermediate or long paxillin dwell times showed that the majority of pixels in images from cells expressing paxillin-S273A (68%) were classified with long dwell times in adhesions while the pixels in images from cells expressing paxillin-S273D were mostly classified with short adhesion dwell times (71%) (Fig. 3C). Paxillin-WT adhesions demonstrated a broader distribution of paxillin binding dynamics (Fig. 3C).

Upon closer inspection of binding dynamics of paxillin-WT across adhesions it became obvious that there were also differential binding dynamics within individual adhesions (Fig. 3D, top panel). In fact, short dwell times or faster dynamics were observed at the membrane proximal ends of adhesions at the protrusive edge of the cell (Fig. 3D). In contrast,  short dwell times were observed at the membrane distal end of the adhesions at the retracting edge of the cell (Fig. 3D, bottom panel). This polarization of dynamics within adhesion subdomains was not apparent with the paxillin phosphorylation mutants suggesting that it depends on differential states of paxillin S273 phosphorylation (Fig. 3B).

To further investigate the paxillin binding rates and their dependence on phosphorylation of S273, pharmacological inhibition or enhancement of paxillin phosphorylation with IPA-3 or okadaic acid, respectively, was explored. IPA-3 treatment resulted in a significant decrease in the paxillin binding rate, resulting in more pixels showing long dwell times (Supplemental Fig. S4A,B). On the contrary, application of okadaic acid led to a significant increase in paxillin binding rates at adhesions (Supplemental Fig. S4C,D).

Collectively, these results illustrate that phosphorylation of paxillin at S273 via PAK1 kinase activity is dependent on βPIX binding to PAK1 and βPIX GEF activity and differentially regulates the binding dynamics of paxillin. Binding that is more dynamic was observed at protrusive edges of the cell and less dynamic binding at retractive edges. In addition, selective paxillin phosphorylation at S273 is required for differential binding dynamics in subregions of individual adhesions with more rapid binding at the growing edge of the adhesions.

### PAK1 and βPIX are enriched in some adhesions and have slower binding dynamics compared to non-adhesion regions

**Figure 4:**
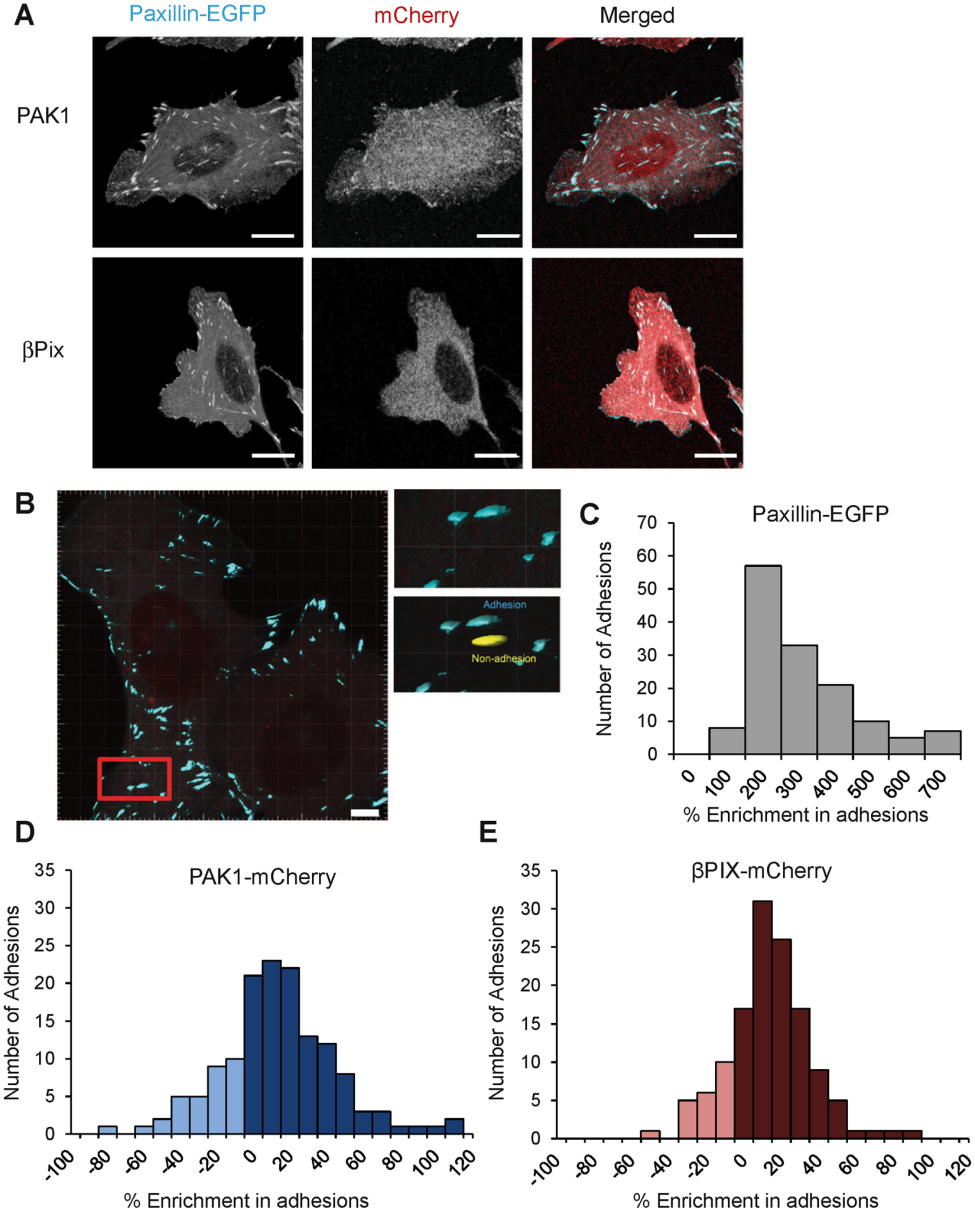
PAK1 and βPIX do not visibly co-localize with paxillin but are enriched in adhesions. (**A**) Laser scanning confocal images of cells co-expressing paxillin-WT-EGFP with transiently transfected βPIX-WT or PAK1-WT mCherry fusions. Scale bar is 10 μm. (**B**) Intensity analysis was performed on adhesions and adjacent non-adhesion areas. Scale bar is 5 μm. The inset illustrates how the analysis was achieved (cyan vs yellow ROIs). Distribution of (**C**) paxillin-WT-EGFP, (**D**) PAK1-WT-mCherry and (**E**) βPIX-WT-mCherry intensity levels binned according to percentage enrichment in adhesions and adjacent non-adhesion areas. Positive values on the histogram (dark blue or dark red) represent enrichment higher in the adhesions and negative numbers denote a higher enrichment in non-adhesion areas (light blue or light red). Paxillin was always enriched in the adhesion versus the non-adhesion areas. n = 130–140 adhesions and non-adhesion regions from at least 10–15 cells.

From experiments presented thus far, it is evident that PAK1 and βPIX play a significant role in the regulation of cell migration and adhesion dynamics. However, laser scanning confocal images reveal that neither of these proteins significantly localize at adhesions marked by paxillin, but they rather have a diffuse cellular distribution (Fig. 4A). To determine if these proteins were indeed enriched in adhesions, the fluorescence intensities of PAK1-WT or βPIX-WT were measured at paxillin-WT containing adhesions and compared to identically-sized adjacent regions of the cell where there were no adhesions (Fig. 4B). Paxillin was always highly localized in adhesions, with percentage enrichments several-fold higher than non-adhesion regions (Fig. 4C). PAK1 and βPIX were indeed localized to many adhesions but with enrichments of only ~5–50% over non-adhesion regions (Fig. 4D,E).

**Figure 5:**
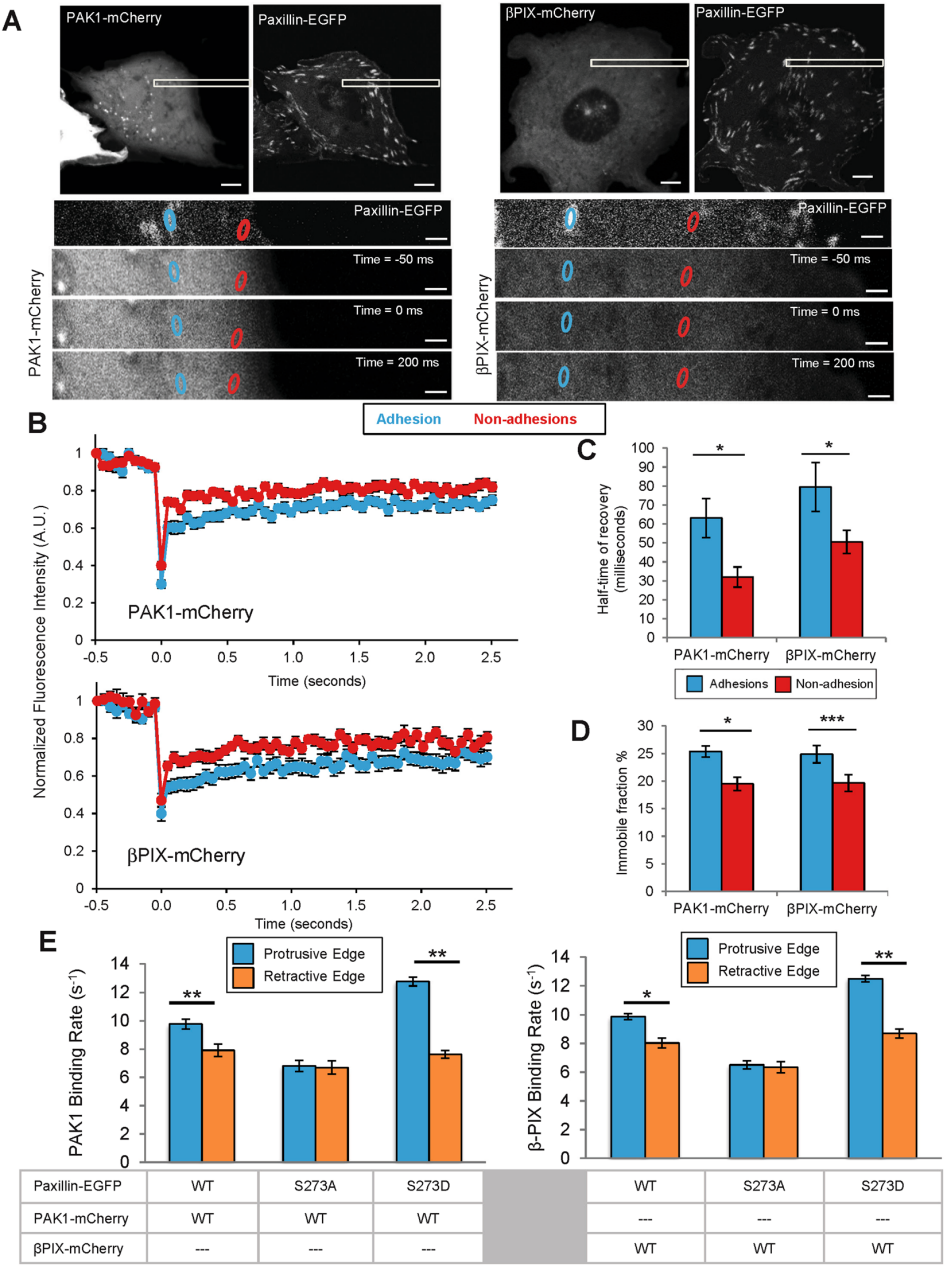
PAK1 and βPIX proteins are less dynamic in adhesions and have a higher immobile fraction. Binding rates at adhesions depend on paxillin pS273. (**A**) Images of cells and FRAP image time series of cells co-expressing paxillin-WT-EGFP and PAK1-WT or βPIX-WT mCherry fusions. Adhesions were identified by paxillin-EGFP localization and mCherry fusions were bleached with a 561 nm laser. Scale bars are 5 μm. (**B**) The recovery curves of PAK1-WT (top) and βPIX-WT (bottom) in adhesions and the non-adhesion regions are shown. Each curve was averaged over multiple adhesions (n = 60 adhesion or non-adhesion ROIs), multiple cells (n = 10–15) and 3 independent experiments. The first time point plotted after bleaching for each curve was obtained from FRAP experiments done on fixed regions since the proteins were already recovered significantly in live cell experiments. (**C**) Half-time of fluorescence recovery and the (**D**) immobile fraction were determined in both adhesion and non-adhesion regions. (**E**) ICM data showing average protein binding rates for PAK1-WT or βPIX-WT in cells expressing with paxillin variants in protrusive (cyan) and retractive (orange) edges of the cell. n = 10–20 cells. All error bars are SEM. For D one star exceptionally corresponds to P < 0.05. In all other cases, one Star (*) corresponds to p < 0.01, two stars **p < 0.001, three stars ***p < 0.0001.

To characterize the dynamics of PAK1 and βPIX in adhesions versus non-adhesion regions of the membrane, FRAP experiments were conducted using a laser scanning confocal microscope (Fig. 5). Adhesions and nearby non-adhesion regions were outlined using the paxillin-WT-EGFP signal since PAK1 and βPIX did not extensively localize to adhesions. The mCherry signal was then bleached with a 561nm-laser and the fluorescence recovery was monitored (Fig. 5A). PAK1 and βPIX exhibited rapid dynamics in both adhesions and non-adhesions regions of the cell such that there was rapid recovery even at the first time point acquired post-bleaching (Fig. 5A,B). Due to the rapid dynamics, the zero time point after bleaching was estimated from control experiments conducted on fixed samples. The FRAP curves from multiple adhesions, cells and experiments were averaged and fit to a single exponential equation to extract recovery times and immobile protein fractions (Supplemental Fig. S5A,B). The half-time recoveries for PAK1 and βPIX were 51% and 38% higher, respectively, in the adhesions than in non-adhesion regions, indicating significantly slower dynamics in adhesions. (Fig. 5C). Quantitative analysis revealed that both PAK1 and βPIX had an immobile fraction of ~25 ± 1% (Fig. 5D) on the timescale of the experiments (2.5 seconds). This was significantly higher than the immobile fraction of ~20 ± 1% in non-adhesion regions (Fig. 5D). These results show that PAK1 and βPIX exhibit fast dynamics in the cell, but have a higher residence time within paxillin-containing adhesions.

### PAK1 and βPIX adhesion binding rates are dependent on paxillin pS273

tICM analysis was conducted to characterize further the binding dynamics of PAK1 and βPIX across the entire cell in the presence of different paxillin mutants. Cells stably expressing paxillin-WT, -S273A or -S273D mutants were transfected with PAK1-WT or βPIX-WT. The paxillin fluorescence signal was used to focus the TIRF microscope on adhesions at the bottom surface of the cell and tICM image acquisition and analyses were performed to map out the binding dynamics of PAK1 and βPIX directly. Half times of binding decays were in the range of 50–150 ms, similar to the FRAP recovery half-times (Fig. 5C). The binding times were converted to protein binding rates ranging from 9 s^−1^ to 20 s^−1^ (Fig. 5E). PAK1 and βPIX demonstrated intermediate binding dynamics when they were co-expressed with paxillin-WT, and faster or slower binding dynamics when they were co-expressed with paxillin-S273D or -S273A, respectively (Fig. 5E). Differential PAK1 and βPIX binding dynamics were measured across the cell with faster dynamics at protrusive edges when co-expressed with paxillin-WT or paxillin-S273D but not with paxillin-S273A (Fig. 5D,E, Supplemental Fig. S6). Interestingly, the binding rates for PAK1 and βPIX are on the same timescale of binding rates obtained with paxillin-WT, which suggests that they are binding and unbinding as part of a mutual protein complex.

### Paxillin/PAK1 and paxillin/βPIX co-bind dynamically to adhesions as a complex

To explore protein complex formation and complex binding dynamics within individual adhesions, temporal Image Cross-Correlation Microscopy (tICCM, also known as image FCCS) was performed. Basically, fluorescence intensity signals from two different fluorescent proteins were collected simultaneously over time on a TIRF microscope with two camera detectors (Materials and Methods). The technique has been reviewed in detail^41^. If the two proteins are bound to one another, either directly or indirectly within a common protein complex, then changes in their fluorescence intensity signals will be correlated when they dynamically bind and unbind at adhesions. A cross-correlation function is calculated from a statistical analysis of the intensity fluctuations of the two fluorescent species. The amplitude of the cross-correlation function is related to the amount of co-binding of the two proteins and the shape of the function reflects the co-binding dynamics.

Control experiments were performed to demonstrate the tICCM technique and ensure that there were no artifacts due to emission crosstalk between the two camera detectors. Crosstalk would show up as 100% cross-correlated. Tensin-mCherry was chosen as a negative control because it localizes to focal adhesions but does not interact with paxillin^44^ (Supplemental Fig. S7A). The positive control involved the expression of a dual paxillin protein fusion, EGFP-paxillin-mCherry (*i.e*., N-terminal EGFP, C-terminal mCherry, Supplemental Fig. S7B). The negative control validated the tICCM analysis and confirmed that there was essentially no fluorescence crosstalk between the two detector channels (Supplemental Fig. S7C, top panel). When the tICCM analysis was applied on cells expressing the dual-labelled paxillin construct, co-binding was detected across all adhesions, as indicated by the red pixels across entire adhesions (Supplemental Fig. S7C, bottom panel).

The interactions between the different paxillin, PAK1 and βPIX wild-type and mutant constructs were biochemically confirmed by co-immunoprecipitation (IP) assays (Supplemental Fig. S8). As shown in the immunoblots, there is no marked difference in the binding of paxillin to any of the PAK1 and βPIX mutant proteins (Supplemental Fig. S8A,B). Note that the expression level of the paxillin-S273A, paxillin-S273D and βPIX-LL mutant was low but still associated with paxillin. The amount of binding of PAK1-WT or βPIX-WT to the paxillin S273A and S273D mutants was not significantly different (Supplemental Fig. S8C). This could result from PAK1 or βPIX binding to populations of paxillin outside of adhesions, binding to other domains on paxillin or due to overall weak binding resulting in little retention of the protein complex with paxillin during IP sample preparation experiments. This emphasizes the need to have techniques that can measure protein interactions *in situ* in the cell.

**Figure 6:**
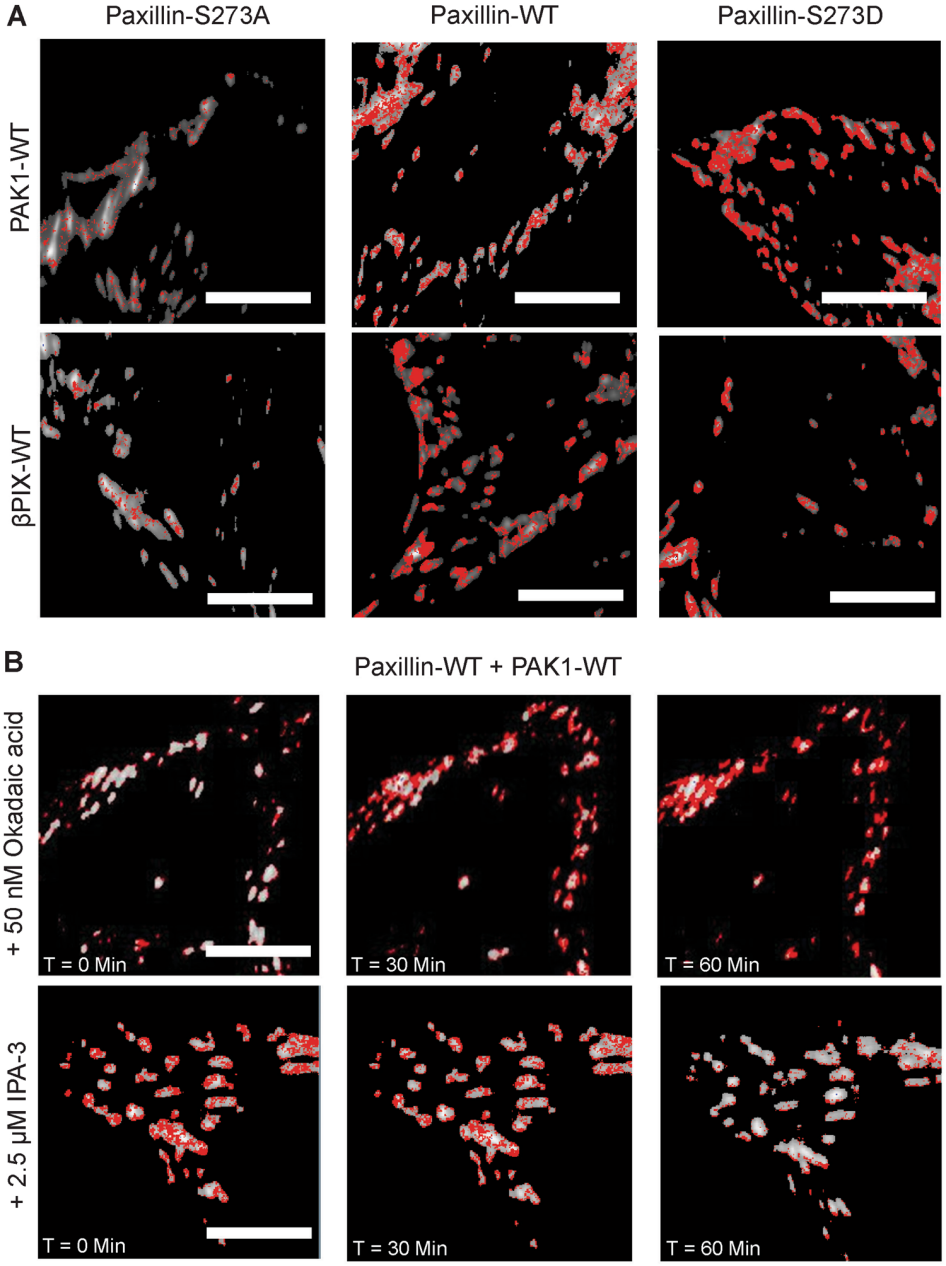
Paxillin phosphorylation state at S273 influences the degree of interaction between paxillin and PAK1 or paxillin and βPIX at sites of adhesion. (**A**) Spatial protein co-dynamic binding maps across cells co-expressing PAK-WT or βPIX-WT mCherry fusions and paxillin-WT, -S273A, or -S273D EGFP fusions. (**B**) Spatial protein co-dynamic maps across cells expressing paxillin-WT-EGFP and PAK1-WT-mCherry before, 30 and 60 minutes after treatment with 2.5 μM IPA-3 (PAK1 inhibitor) or 50 nM Okadaic acid (PP2A phosphatase inhibitor). Scale bars are 5 μm. Red pixels indicate where the two proteins are co-binding.

Measurements of co-binding dynamics using the tICCM analysis were performed on cells co-expressing paxillin-EGFP and either PAK1- or βPIX-mCherry. The results generated, for the first time, whole-cell spatial maps showing that paxillin-WT was co-binding with PAK1-WT and βPIX-WT in an S273 phosphorylation dependent manner (red pixels, Fig. 6A,B). Little co-binding was seen in cells expressing the phospho-deficient paxillin-S273A mutant and high co-binding was observed with expression of paxillin-S273D (Fig. 6A,B).

Next, the levels of paxillin’s interaction with PAK1 or βPIX in the presence of IPA-3 or okadaic acid was assayed with immunoprecipitation experiments. Cells expressing paxillin-WT were transfected with PAK1-or βPIX-mCherry and then treated with one of the pharmacological inhibitors (Supplemental Fig. S8D–G). While the amount of PAK1 immunoprecipitated with paxillin following OA treatment was unchanged, the amount of βPIX increased significantly (Supplemental Fig. S8D,F). No significant changes in PAK1 or βPIX association with paxillin were observed when PAK1 activity was inhibited with IPA-3 (Supplemental Fig. S8G). A point of interest is that our immunoprecipitation studies did not show evident changes in the levels of protein association between the various paxillin S273 mutants, and the βPIX and PAK1 constructs. Thus, lack of paxillin S273 phosphorylation did not completely abrogate the association of βPIX and PAK1 with paxillin. Paxillin kinase linker (PKL), a protein related to GIT1, has been previously reported to bind to the LD4 motif of paxillin and serve as another mechanism for βPIX and PAK1 recruitment to adhesions^45–47^. The influence of this signaling pathway in CHO cells cannot be excluded since overexpression of a βPIX-deficient in GIT1-binding show no difference in migration rates and adhesion dynamics^10^. Thus, the PKL/βPIX/PAK1 pathway could compensate for βPIX’s inability to bind to GIT1 by promoting its interaction with PKL.

To determine how the dynamics of the paxillin/PAK1 interactions were affected at focal adhesions, tICCM was conducted in the presence of IPA-3 or okadaic acid. This permitted real-time spatial and temporal identification of the location of co-binding. When PAK1 kinase activity was inhibited with IPA-3, paxillin/PAK1 co-binding was quickly diminished and this reduction could be followed over time (Fig. 6B). In turn, when paxillin dephosphorylation was inhibited by OA, paxillin/PAK1 co-binding interactions were increased (Fig. 6B). Interestingly, the co-binding was maximal around the edges of the adhesions after 60 minutes, suggesting perhaps that the proteins in the middle of the adhesions are less mobile while proteins at the edges of the adhesions are dynamic. These tICCM experiments also demonstrate that determining the absolute levels of interaction between paxillin and PAK1 by IP is insufficient to appreciate the localized changes in the dynamics of these proteins at focal adhesions upon treatment with pharmacological agents.

**Figure 7:**
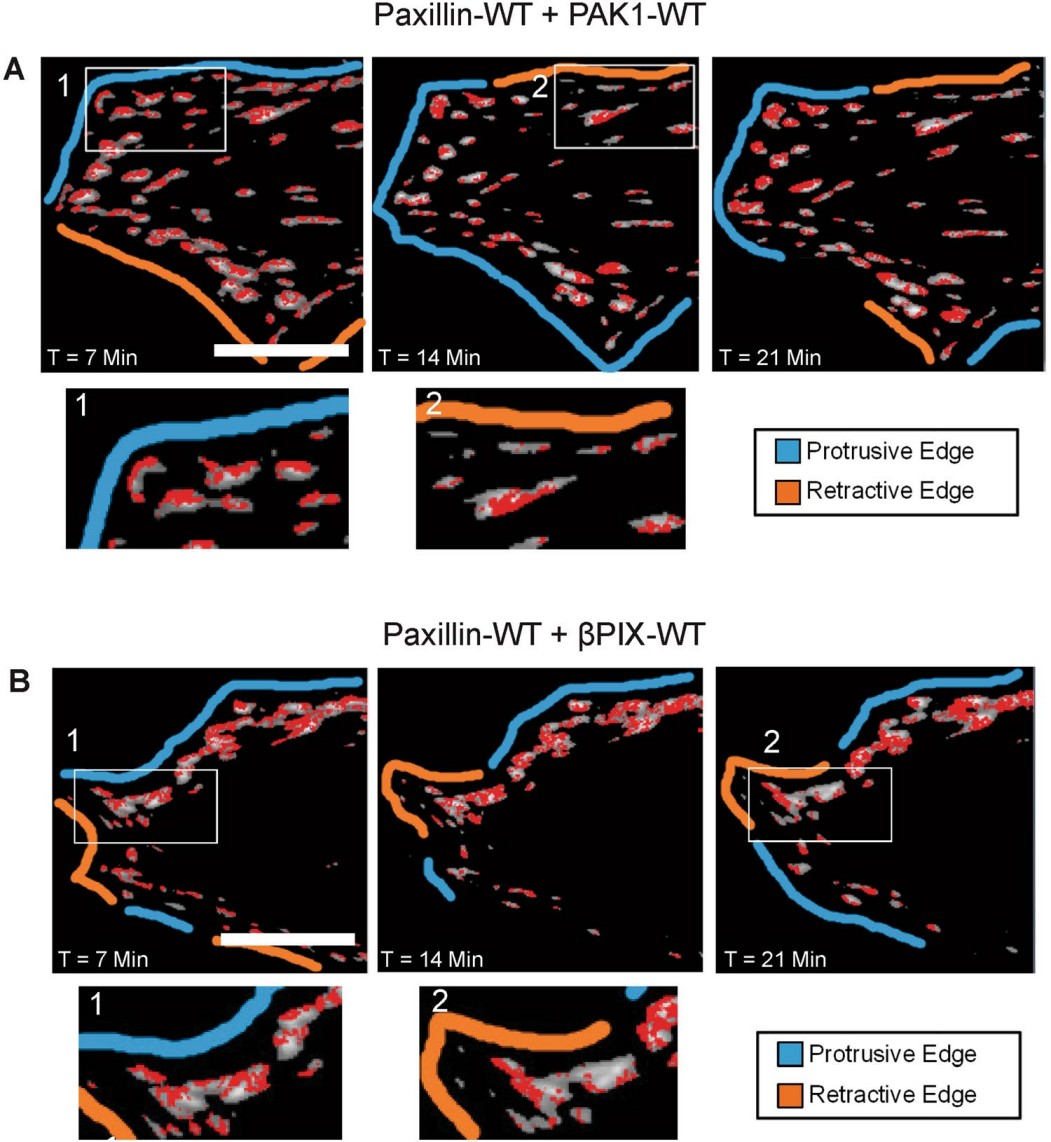
Paxillin co-binding with PAK1 or βPIX occurs at new dynamics edges of adhesions. Spatial protein binding maps of cells co-expressing paxillin-WT-EGFP and (**A**) PAK1-WT-mCherry or (**B**) βPIX-WT-mCherry. Red pixels indicated areas of co-binding. Co-binding occurs proximal to protrusive cell edges (cyan lines) and distal from retractive cell edges (orange lines) as shown in enlarged areas indicated by white boxes. Cells were imaged over time and as protrusive and retractive regions changed, so did the location of co-binding. Scale bars are 5 μm.

Further investigation of co-binding at protrusive and retractive edges of the cell was conducted. Each cell was imaged for seven minutes prior to tICCM image collection to identify protrusive and retractive edges. Dual-colour time-lapse images were collected for 1–2 minutes for tICCM analysis in areas where paxillin was localized to adhesions. Most interestingly, paxillin-WT showed variable co-binding with PAK1-WT and βPIX-WT across individual adhesions (Fig. 7A,B). This polarized interaction within adhesion subregions was lost with paxillin-S273D as interactions occur across the entire adhesion regardless of location in the cell (Fig. 6A). The co-binding seemed to be selective for the same dynamic regions of the adhesions identified with tICM; that is, the distal tip near the protrusive edges (blue line) of the cell and the proximal tip (side of elongated adhesions parallel to the retracting edge) of adhesions away from the membrane at retracting edges (orange lines) of the cell (Fig. 7A,B). In fact, in some areas of the cell, the protein interaction was detected initially at the protrusive edge (enlarged area 1, time zero to seven minutes) and later in the retracted edge (enlarged area 2 after approximately twenty-one minutes) (Fig. 7A,B). In both cases shown, the subregions of co-binding of paxillin with PAK1 (Fig. 7A) and βPIX (Fig. 7B) shift from the membrane-proximal region of adhesions when the edge was protrusive (enlarged area 1) to the membrane-distal regions of the adhesions as the cell retracted (enlarged area 2). Consistent with the individual protein tICM measurements the co-binding rates for paxillin/PAK1 or paxillin/βPIX were similar (10 s^−1^) to measurements for each individual protein (Figs 3A and 5E).

## Discussion

Cell migration is a complex process that involves spatial and temporal regulation of protein localization and dynamics across the cell. This regulation is related to the spatial localization, dynamics, size and lifetime of adhesions linking the cytoskeleton to the extracellular matrix. This study looked at cell migration from the whole cell down to the sub-microscopic level of protein binding and co-binding at individual adhesions. The results here solidify the key role of paxillin phosphorylation at S273 as a switch that controls cell migration by regulating adhesion dynamics^10^. We demonstrate that regulation of adhesion dynamics via paxillin S273 phosphorylation involves intricate changes in protein binding rates and intermolecular interactions within adhesion subdomains. PP2 phosphatase activity, PAK1 kinase activity, and βPIX binding to PAK1 and its GEF activity all play a role in this regulation.

This study generated a large amount of quantitative data at the cellular, adhesion and protein complex levels. This data will be valuable for the development of new computational models of cell migration and adhesion. In fact, we have already used the data to develop a new model that has helped us to further understand the role of paxillin S273 phosphorylation and its effects on the small GTPases Rac1 and RhoA in regulating adhesion dynamics^48^. In this study, we identified two paxillin-WT adhesion subpopulations experimentally: one was small dynamic adhesions and one was larger stable adhesions^48^. This bimodality was abolished experimentally with expression of paxillin-S273D or S273A mutants, which had predominantly small, dynamic or large, stable adhesions, respectively^48^. The model also identified a bistable switch consistent with the two populations of adhesions, dynamic or stable. The model showed that this bistability favored dynamics adhesions when PAK1 activation was high, and stable adhesions when PAK1/βPIX binding was low^48^. The switch from stable to more dynamic adhesions is likely dependent on Rac1 activation at adhesions through paxillin S273 phosphorylation and localization of βPIX and PAK1. Overall, the model is consistent with the results in this study and can be further refined based on the extensive quantitative data that has been generated. For example, we will explore the role βPIX GEF activity plays in maintaining bistability of adhesions.

βPIX has exchange factor activity for both Rac1 and Cdc42 but the activation of these small GTPases is likely locally restricted, dependent on cellular context and variable for different cell types. Cdc42 has been reported to be involved in different activities related to cell migration such as filopodia formation as well as re-orienting microtubules and organelles such as the Golgi apparatus in the direction of migration^49^. Our studies strongly support a function for βPIX specificity towards Rac1 activation because expression of a dominant-negative Rac1 was able to inhibit significantly rapid focal adhesion dynamics in cells expressing paxillin-S273D^10^. However, we can’t rule out a similar role in Cdc42 activation here, but it is likely that Rac1 is dominate in CHO cells as they do not demonstrate filipodia-like structures.

A key finding in the study was the consistent differential in adhesions dynamics at the protrusive versus retractive edges of the cell. It is probable that adhesion assembly and disassembly are regulated at the molecular level through variable paxillin binding kinetics as was measured with tICM. This distinction in adhesion dynamics and protein binding at the different cell edges were dependent on paxillin S273 phosphorylation, PAK1 kinase activity and βPIX localization and GEF activity. At protrusive edges, paxillin S273 phosphorylation would be relatively high leading to more rapid binding and more dynamic adhesions. This dynamic binding would be maintained by PAK1 binding to βPIX and the localization of this protein complex to adhesions via GIT1. This would lead to the feedback mechanism proposed by Nayal et. al. and high local Rac1 activity, more active PAK1 and high paxillin S273 phosphorylation. This idea is supported by the observation that PAK1 and βPIX are enriched in paxillin-containing adhesions and show longer residence times. If this cascade is interrupted through inhibition of PAK1 or βPIX activity, or by lack of localization of the complex to adhesions, then local phosphorylation of paxillin S273 is inhibited and the adhesion can stabilize. PAK1 is absent in the adhesions of cells with high RhoA activity^50^ and RhoA is usually activated at retractive edges of the cell, where it might promote low levels of localized paxillin S273 phosphorylation^49^. It was somewhat surprising to see this differential in adhesion dynamics maintained in the presence of paxillin-S273D or PAK1-CA overexpression as one might have expected rapid adhesion dynamics across the entire cell. This suggests factors downstream of paxillin S273 phosphorylation are likely at play and maintain distinct adhesion assembly and disassembly kinetics at the cell front and rear.

A notable observation from our studies is that the binding kinetics of exogenously expressed fluorescent paxillin across individual adhesions is variable, with shorter binding times near the membrane proximal edge of the adhesion in protrusive areas of the cell. This rapid binding is likely regulated by paxillin S273 phosphorylation as pharmacological interventions show shorter binding times when phosphatases are inhibited and longer binding times when PAK1 kinase activity is inhibited. Adhesion subdomains are only observed if paxillin S273 phosphorylation can be regulated as they are not observed with the paxillin-S273A or -S273D mutants. The most novel finding was that subdomains of the adhesion with rapid paxillin binding correspond to regions where paxillin/PAK1 or paxillin/βPIX co-bind to the adhesion as a complex. This co-binding depends on paxillin S273 phosphorylation and PAK1 kinase activity.

**Figure 8:**
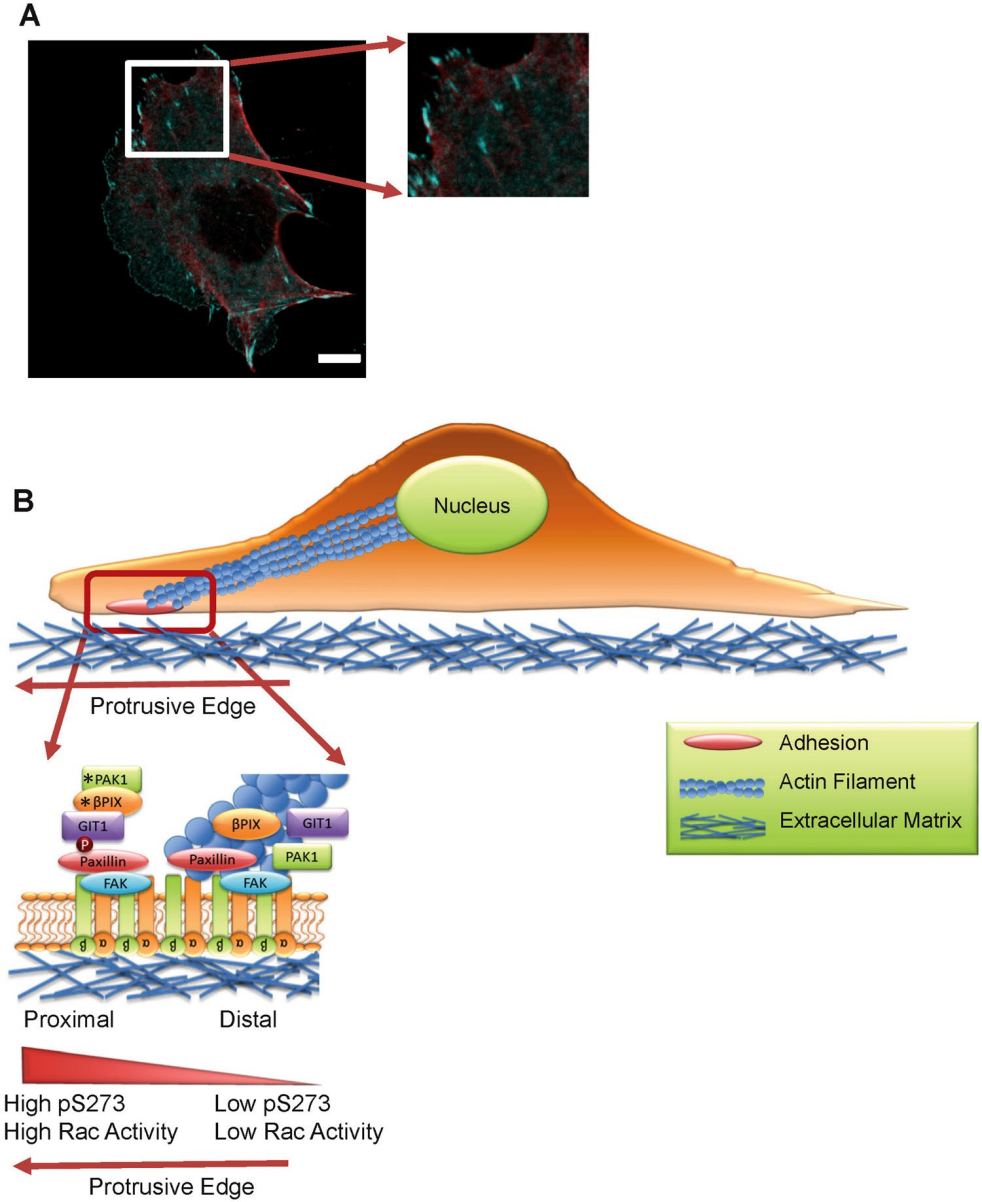
Proposed model of effects of paxillin S273 phosphorylation on adhesion binding, regulation of adhesion dynamics and cell migration. (**A**) Protrusive region of CHO-K1 cell expressing paxillin-S273A-EGFP (cyan) and actin stained with phallodin AF594 (red) indicating actin cables interacting with the membrane distal region of adhesions. (**B**) Proposed model of spatial coordination of paxillin pS273 and binding to the PAK1 and βPIX through GIT1 at the membrane proximal end of adhesions in protrusive regions of the cell. The model proposes that localization of paxillin pS273 bound with PAK1 and βPIX to membrane proximal regions of the adhesion is associated with higher Rac GTPase activity, active PAK1 kinase activity, βPIX GEF activity and minimal interactions with the actin cytoskeleton.

Previous work has shown that paxillin exists as a monomeric protein in the cytosol of CHO-K1 cells and binds to assembling adhesions as a monomer^40^. In addition, paxillin is only seen as a larger protein complex when leaving adhesions^51^. Taken with the results presented here, this suggests that paxillin, PAK1 and βPIX localize within adhesions to form a complex that regulates adhesion dynamics. That complex then likely leaves the adhesion intact. So the working model is that paxillin is phosphorylated on S273 in the newly assembling dynamic part of the adhesion, which corresponds to the membrane proximal region of adhesions localized near the protrusive edge of the cell (Fig. 8). This would be consistent with the protein complex paxillin/PAK1/βPIX being localized to these subdomains as is seen with the tICCM data. Wolfenson *et al*.^43^ did not observe changes in the juxtaposed paxillin population binding rates across adhesions, perhaps because they were looking at non-migratory cells in steady state. If high paxillin S273 phosphorylation is maintained, PAK1 will remain active, βPIX GEF activity will stimulate Rac1 activity, the cell will continue to protrude, and the adhesion will rapidly disassemble. If paxillin S273 is dephosphorylated, the paxillin/PAK1/βPIX complex will dissociate, Rac1 activity will decrease and the adhesion will associate more tightly with the actin cytoskeleton and stabilize (Fig. 8). Based on these adhesion subdomains, focal complexes could have a dual nature and processes that stabilize or destabilize either subdomain could tilt the balance towards adhesion disassembly or stabilization. For example, stabilization could be due to local myosin IIA recruitment by PAK1, followed by force generation promoting dissociation of PAK1 and reduced paxillin phosphorylation^31^.

Previous studies have shown increased paxillin phosphorylation on Y31 and Y118 by focal adhesion kinase (FAK) within adhesion subdomains^52^. In this case, paxillin Y31/Y118 tyrosine phosphorylation was elevated at the membrane distal end of adhesions in retracting areas of the cell^52^. We speculate that perhaps paxillin phosphorylation at different residues could be regulating interactions between adhesions and the actin cytoskeleton and thus regulating adhesion dynamics and stability. In fact, the LD4 domain on paxillin is a known binding site for both FAK and GIT1. Phosphorylation of S273 within the LD4 domain of paxillin by PAK1 enhances binding to GIT1, and reduces binding to FAK^10,53,54^. In turn, FAK phosphorylates both Y31 and Y118^55,56^ on paxillin and, as mentioned above, this occurs at the opposite end of the adhesion from S273 phosphorylation. Perhaps competitive binding of GIT1 and FAK to the LD4 domain regulates paxillin phosphorylation on Y31/Y118 or S273, which in turn regulates adhesion dynamics and stability. In protrusive areas of the cell, PAK1 could phosphorylate paxillin at S273 resulting in binding to GIT1. The resulting GIT1/βPIX/PAK1 complex would elevate Rac1 activity, bringing more PAK1 to the membrane proximal tip of adhesions and the maintenance of high paxillin pS273 levels. In turn, at the membrane distal end of the adhesion, the LD4 motif could be associated with FAK resulting in phosphorylation of Y31/Y118 on paxillin, increased paxillin binding to vinculin^57^, and stabilization of adhesion via strengthening of the linkage to the actin cytoskeleton. FAK binding to the membrane distal tip of adhesions at protrusive regions of the cells could be regulated by myosin based tension^57^, variable integrin density within the adhesion^58^ or perhaps localized calcium-dependent calpain cleavage of FAK^59^.

In turn, paxillin Y31/Y118 phosphorylation has been shown to be a requirement for S273 phosphorylation in smooth muscle cells and play a role in regulating actin branching^28^. Perhaps paxillin phosphorylation at all of these sites (i.e., Y31/Y118 and S273) is required to maintain dynamic adhesions, membrane protrusion and cell migration.

These same arguments can be made for subregions within adhesions at retractive regions of the cell, but with the membrane distal end being more dynamic. This is supported by the fact that paxillin is known to undergo more rapid binding at the membrane distal edge of sliding adhesions^40^. Wolfenson *et al*.^43^ and Webb *et al*.^60^ also measured faster paxillin binding dynamics at the membrane distal end of long adhesions in retracting regions of the cell, consistent with the idea that this subdomain of adhesions is highly dynamic and may not be as tightly associated with actin filaments^43,60^.

The data presented here highlights how challenging it can be to perform FRAP experiments to measure rapid protein dynamics in the cell. The protein recovery is nearly complete before the first image post bleaching can be collected. In addition, the dynamics of only one or two adhesions at a time can be measured. Although the FRAP dynamic measurements matched the tICM results well, the lack of a requirement for high laser light for photobleaching and the spatial mapping capability of tICM and tICCM make correlation microscopy a much superior technique. These techniques are really ideal biophysical tools for quantitative light microscopy study of protein dynamics and interactions *in situ*. With their continued development and the application of mathematical modelling, we will continue to elucidate the mechanisms that control cell migration through the regulation of protein binding to control adhesion dynamics and cell migration. We demonstrate differential protein dynamics at protrusive and retractive parts of the cell and the presence of dynamic adhesion subdomains that depend on paxillin S273 phosphorylation. We map out the co-binding of paxillin/PAK1 and paxillin/βPIX in these dynamic adhesion subdomains for the first time. Thus we have solidified the role of paxillin S273 phosphorylation as a switch that regulates cell migration through PAK1 and βPIX.

## Supplementary information

Supplemental Information
