# Supplementary material for: Paxillin S273 Phosphorylation Regulates Adhesion Dynamics and Cell Migration through a Common Protein Complex with PAK1 and βPIX
Source: Sci Rep. 2019 Aug 7;9:11430. doi: 10.1038/s41598-019-47722-3 (PMC6686007; doi:10.1038/s41598-019-47722-3)
Supplement: Supplementary file 1 — Supplemental Information [file 41598_2019_47722_MOESM1_ESM.pdf]

Paxillin S273 Phosphorylation Regulates Adhesion Dynamics and Cell Migration through a Common Protein Complex with PAK1 and βPIX

Abira Rajah[1,†], Colton G. Boudreau[1,†], Alina Ilie[1], Tse-Luen Wee[1,2], Kaixi Tang[1], Aleksandar Z. Borisov[1], John Orlowski[1,4] and Claire M. Brown [1,2,3,4,5*]

1) Department of Physiology, McGill University
2) Advanced BioImaging Facility (ABIF) McGill University, 3649 Prom. Sir William Osler, Bellini Building Rm137, Montreal, QC, Canada, H3G 0B1
3) Department of Anatomy and Cell Biology, McGill University
4) Cell Information Systems (CIS), McGill University
5) Centre for Applied Mathematics in Bioscience and Medicine (CAMBAM), McGill University

† Equal contributions.
* Corresponding Author: claire.brown@mcgill.ca

Short Title: Paxillin regulates adhesion dynamics within sub-domains.

Supplemental figure captions:

Supplemental Figure S1: Cell migration phenotypes of CHO-K1 cells co-expressing paxillin and various PAK1 and βPIX constructs. Rose plots of CHO-K1 cells expressing (A) paxillin WT EGFP and various PAK1 mutant constructs, and (B) paxillin WT EGFP and various PIX mutant constructs.

Supplemental Figure S2: Adhesion disassembly rates are different at the leading and trailing edges of cells. Adhesion disassembly rates for the leading edge of CHO-K1 cells expressing various constructs or combinations of tagged proteins as the leading edge (blue bars) and trailing edge (orange bars).

Supplemental Figure S3: Paxillin S273 phosphorylation induces a short-binding of paxillin population at adhesions. For cells expressing EGFP-tagged paxillin-WT, S273A and S273D, shown are the **(A)** adhesion spatial dynamic maps, **(B)** the corresponding color-coded binding rate histograms and (**C)** the binding rate of histograms normalized to the highest pixel frequency. Cyan pixels represent short-binding (rates 12-20 s^{-1}), yellow for intermediate dynamics (9-12 s^{-1}), and magenta representing long binding (<9 s^{-1}).

Supplemental Figure S4: Pharmacological studies reveal the requirement of paxillin S273 phosphorylation for controlling paxillin's binding rate at adhesions. Paxillin binding rates were quantified after treatment with **(A)** IPA-3 or DMSO or **(C)** okadaic acid or H_2O normalized relative to before treatment of the drug or vehicle. Representative spatial dynamic maps of adhesions and exponential histograms of the binding rates in cells expressing paxillin-EGFP before and after treatment with **(B)** 2.5 μM IPA-3 or **(D)** 50 nM okadaic acid. Cyan pixels represent short-binding (rates 12-20 s^{-1}), yellow for intermediate dynamics (9-12 s^{-1}), and magenta representing long binding (<9 s^{-1}). Scale bar for images are 5 μm. Error bars represent SEM. One-star (*) corresponds to $p<0.05$, two stars (**) $p<0.001$. Three independent experiments and at least 5-10 cells were in each trial.

Supplemental Figure S5: The dynamics of PAK1 and βPIX differ between different cellular regions. Representative recovery of **(A)** PAK1-mCherry and **(B)** βPIX-mCherry localized in adhesion (left) or non-adhesion (right) regions from one FRAP experiment. The data points were overlaid with a simple exponential curve as indicated by the solid line.

Supplemental Figure S6: Intensity profile of CHO-K1 cells expressing paxillin-EGFP with S273 mutations and βPIX-WT-mCherry. Relative amounts of βPIX-mCherry recruited to each region of the cell are represented in cells expressing either paxillin-S273D-EGFP **(A)** or S273A **(B)**. Green arrows indicate the direction of migration of each cell. The donut chart indicates the relative proportion of short (cyan), intermediate (yellow), and long (magenta) binding for the protrusive and retractive edge of the cell. The value displayed in the center of each donut chart indicates the average binding rate for the region analyzed. Scale bar for images are 5 μm.

Supplemental Figure S7: Cross correlation analysis of positive and negative controls. **(A)** Paxillin WT EGFP and tensin mCherry constructs were co-expressed to serve as the negative control. **(B)** A dual labeled paxillin with N-terminal EGFP and C-terminal mCherry was designed as a positive control. **(C)** Spatial co-dynamic maps are shown for CHO-K1 cells expressing either the negative (top panel) or positive control (bottom panel) imaged over time. Scale bar for images are 5 μm.

Supplemental Figure S8: Immunoprecipitation study of PAK1 and βPIX interactions with paxillin. CHO-K1 cells stably expressing paxillin-WT-EGFP were transfected with mCherry-tagged **(A)** PAK1-WT, constitutively-active (CA) and kinase-dead (KD) mutants or **(B)** βPIX-WT and LL. Paxillin was immunoprecipitated with an anti-GFP antibody and were immunoblotted with an anti-mCherry antibody (upper left panels). The membrane was stripped and reblotted with an anti-paxillin (positive control; middle left panels) and anti-tubulin (negative control; lower left panels). To verify expression of transfected constructs, total cell lysates were also immunoblotted with the aforementioned antibodies (right panels). **(C)** Cells stably expressing EGFP-tagged paxillin WT, S273A or S273D mutants were transfected with PAK1- or βPIX-mCherry**.** Cells were lysed and 0.5 mg protein for paxillin-WT and 1 mg protein for mutant paxillin were immunoprecipitated with an anti-GFP antibody or an isotype-matched control IgG antibody, followed by immunoblotting with an anti-mCherry antibody to probe for PAK1-mCherry (top, upper panel) or βPIX-mCherry (top, lower panel). The bottom three panels show total cell lysates expression of the transfected and paxillin-EGFP constructs. Cell lysates were immunoblotted with anti-mCherry (bottom, upper panel) and anti-GFP (bottom, middle panel) antibodies. The blots were stripped and re-blotted with an anti-tubulin antibody to ensure equal loading of protein was achieved for each condition (bottom, lower panel). Cells co-expressing paxillin-EGFP with PAK1 or βPIX-mCherry were treated with **(D)** 100 nM okadaic acid (OA) or **(E)** 10 μM IPA-3**.** Control treatments were with H_2O and DMSO, respectively. Paxillin was immunoprecipitated with an anti-GFP antibody, followed by immunoblotting with an anti-mCherry antibody (upper left panels). Cell lysates were immunoblotted with the anti-mCherry antibody (upper right panels). The blots were stripped and re-blotted with an anti-paxillin and anti-tubulin antibody to reveal the level of immunoprecipitated (lower left panels) and total paxillin and tubulin (lower right panels). The relative amounts of PAK1 and βPIX bound to paxillin were quantified after treatment with OA **(F)** and IPA-3 **(G)** normalized to control treatment. Data are shown as average of three independent experiments. Error bars represent SEM. One star (*) corresponds to $p < 0.05$. The full-length blots from **(A-E)** are presented in Supplemental Figure S9.

Supplemental Figure S9: Full-length (uncropped) immunoblots. **(A)** Supplemental Figure S8A. **(B)** Supplemental Figure S8B **(C)** Supplemental Figure S8C **(D)** Supplemental Figure S8D **(E)** Supplemental Figure S8E. The portion of the blot used in Supplemental Figure S8 are indicated by a red box.

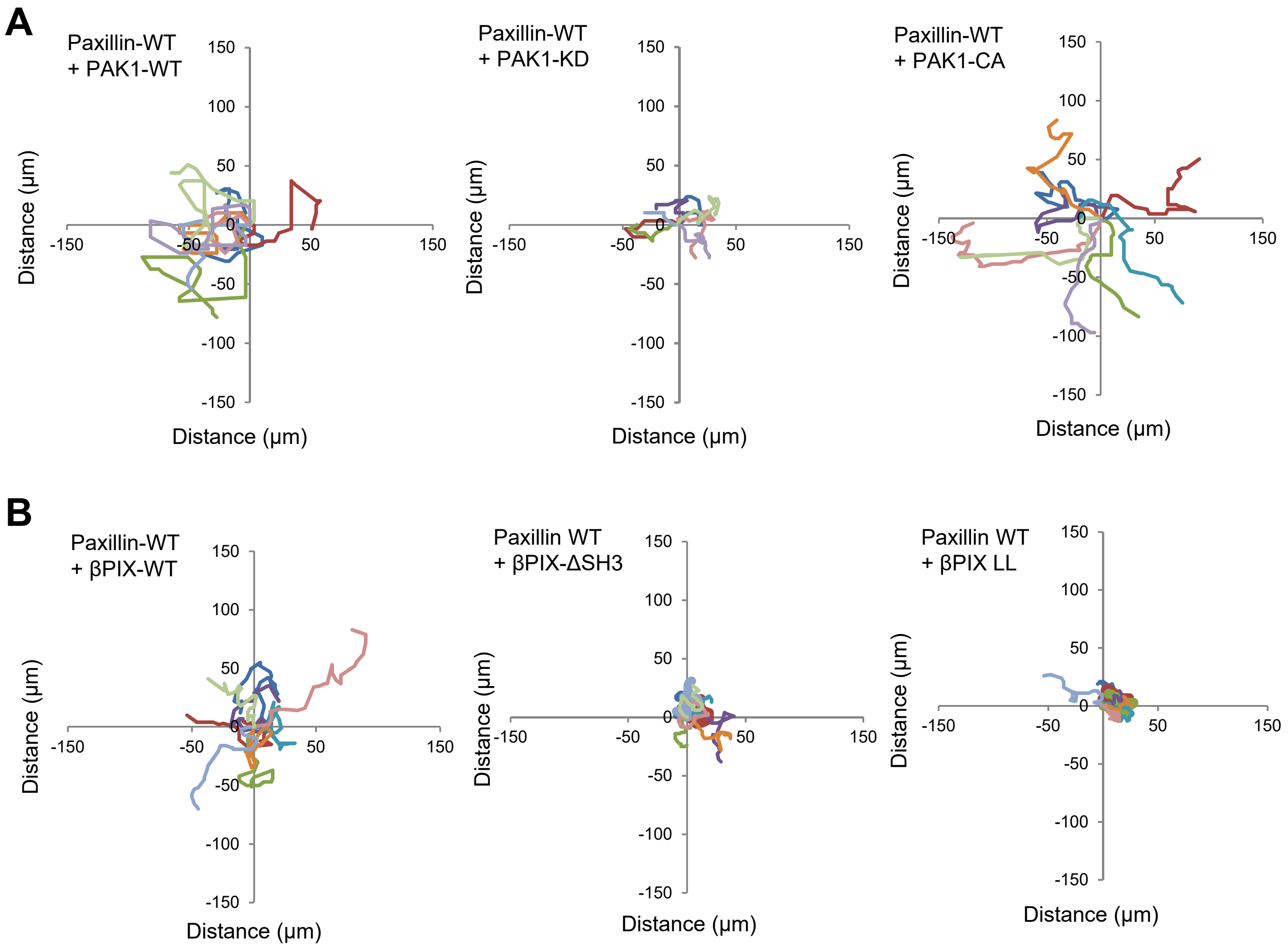

Rajah and Boudreau et al. Supplemental Figure S1

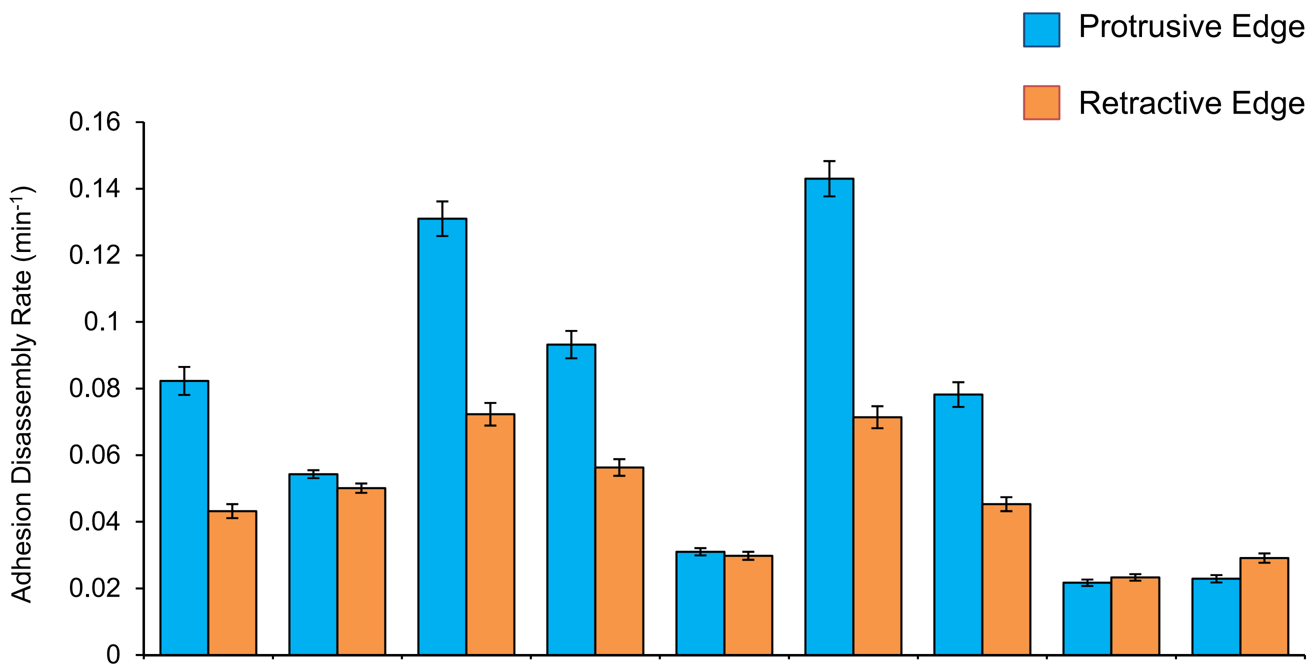

Paxillin	WT	S273A	S273D	WT	WT	WT	WT	WT	WT
PAK1	---	---	---	WT	KD	CA	---	---	---
βPIX	---	---	---	---	---	---	WT	ΔSH3	LL

Rajah and Boudreau et al. Supplemental Figure S2

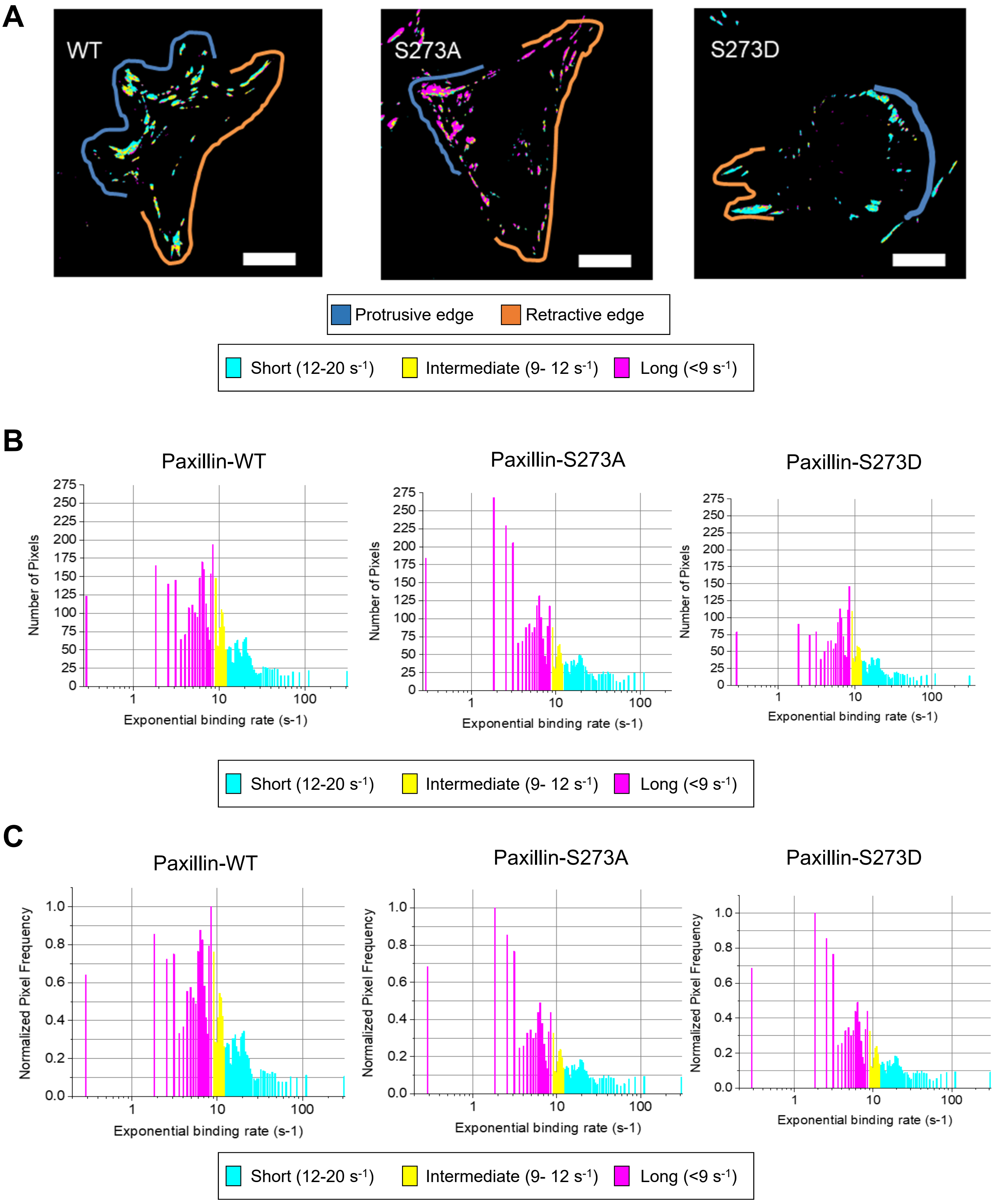

Rajah and Boudreau et al. Supplemental Figure S3

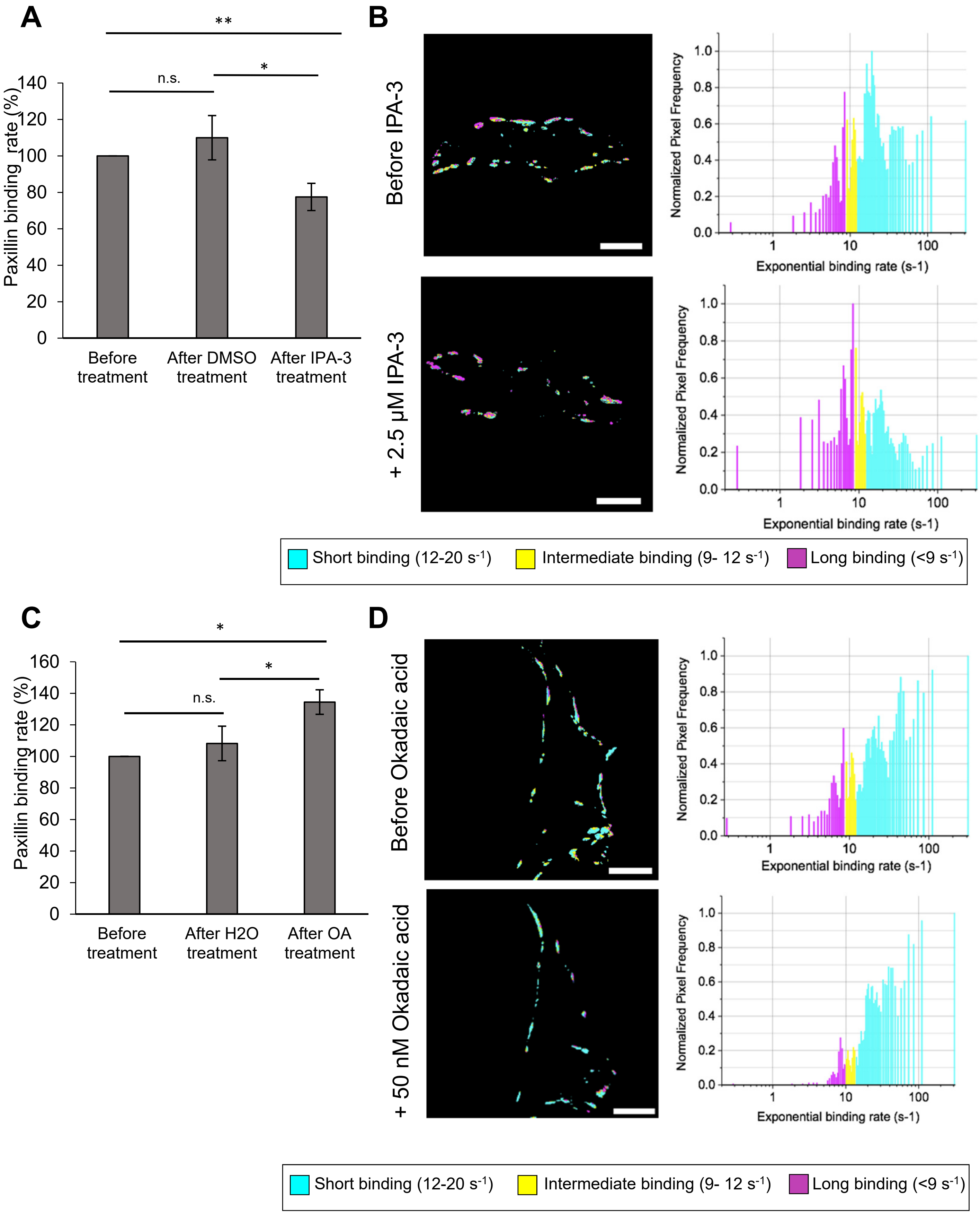

Rajah and Boudreau et al. Supplemental Figure S4

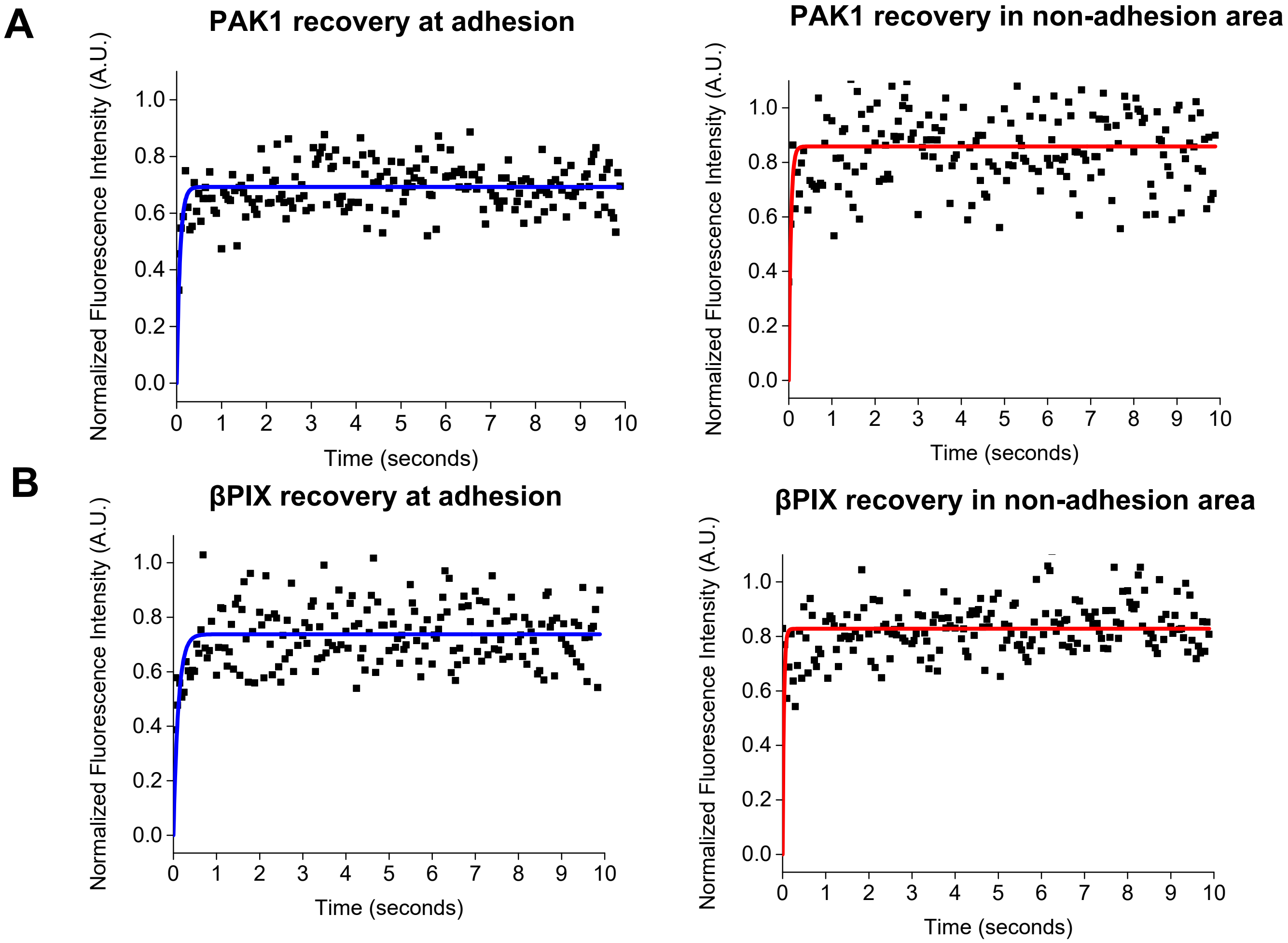

Rajah and Boudreau et al. Supplemental Figure S5

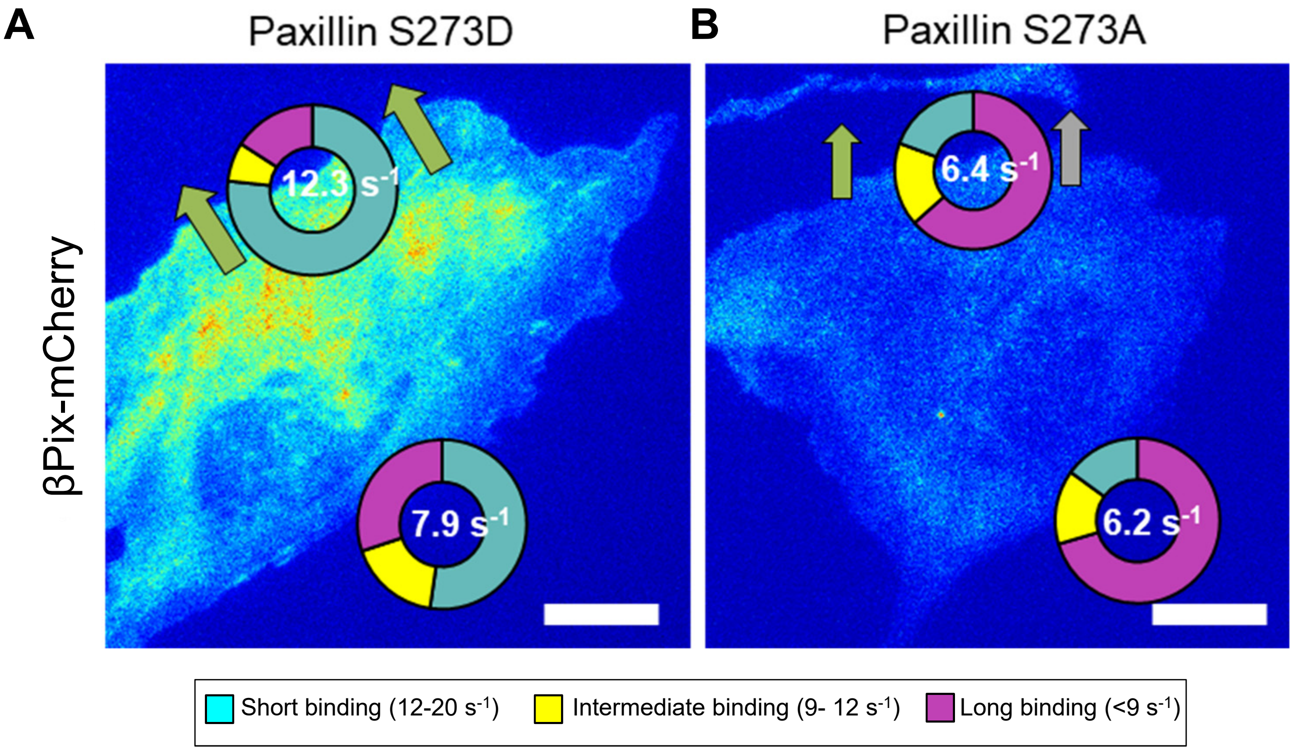

Rajah and Boudreau et al. Supplemental Figure S6

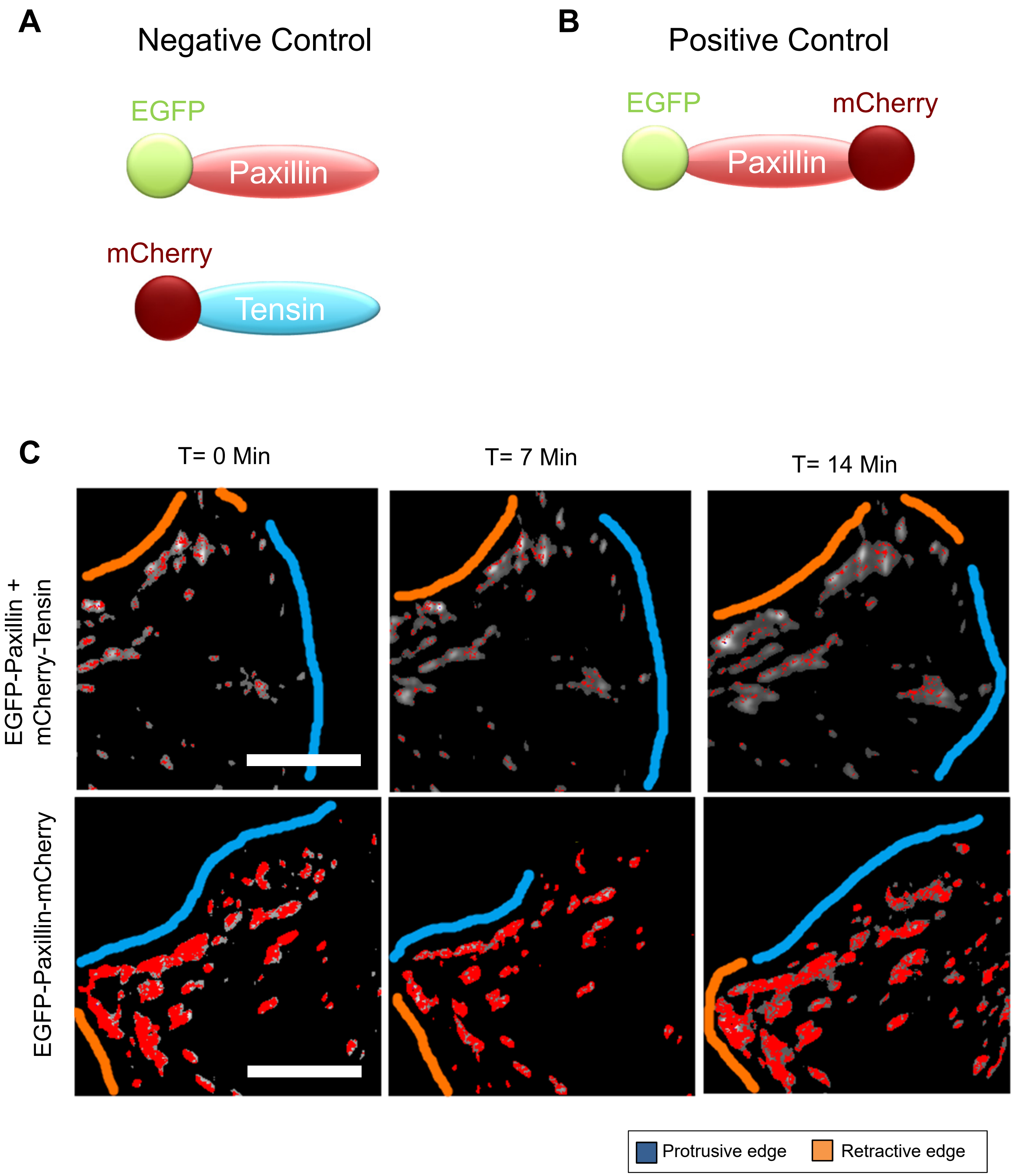

Rajah and Boudreau et al. Supplemental Figure S7

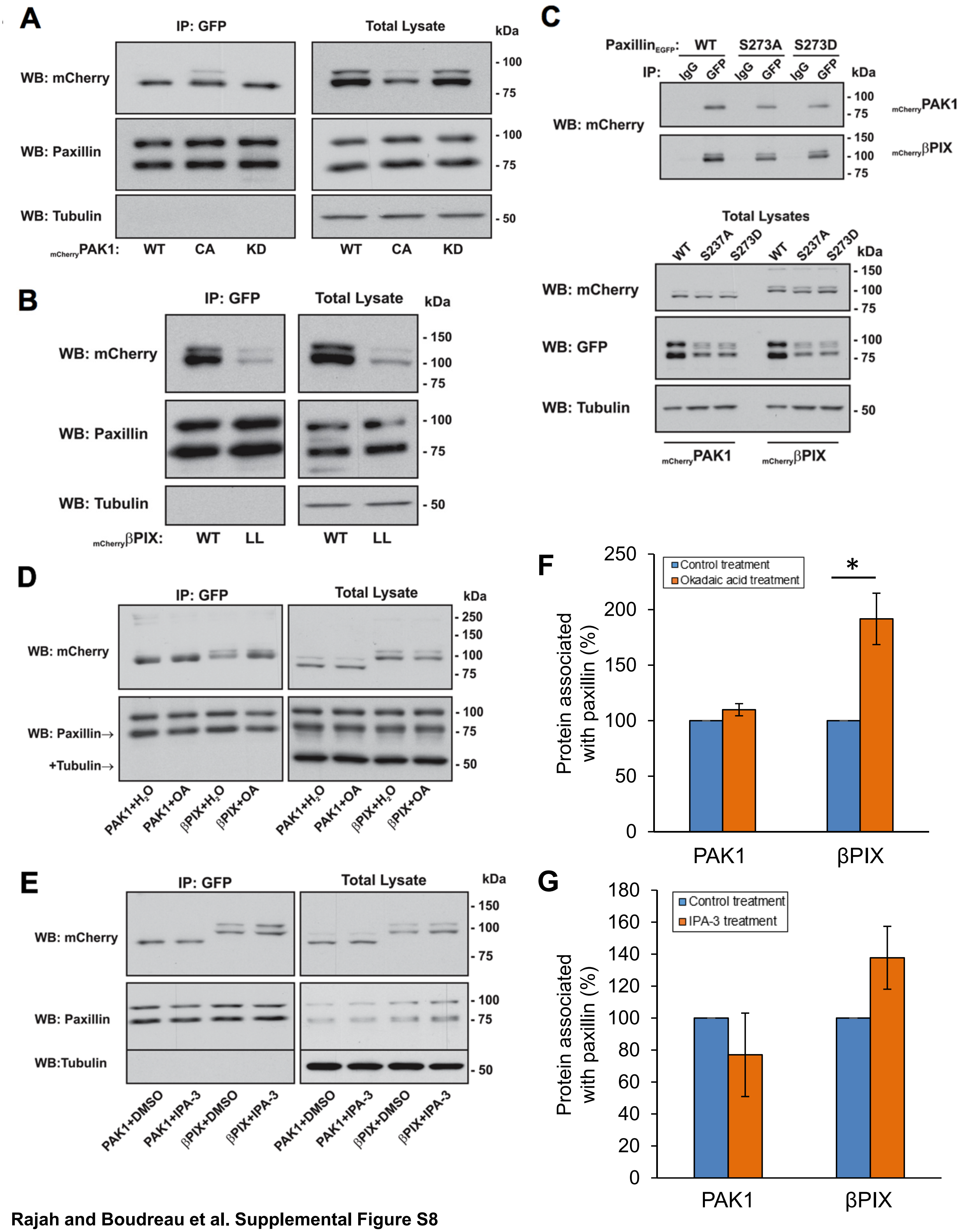

Rajah and Boudreau et al. Supplemental Figure S8

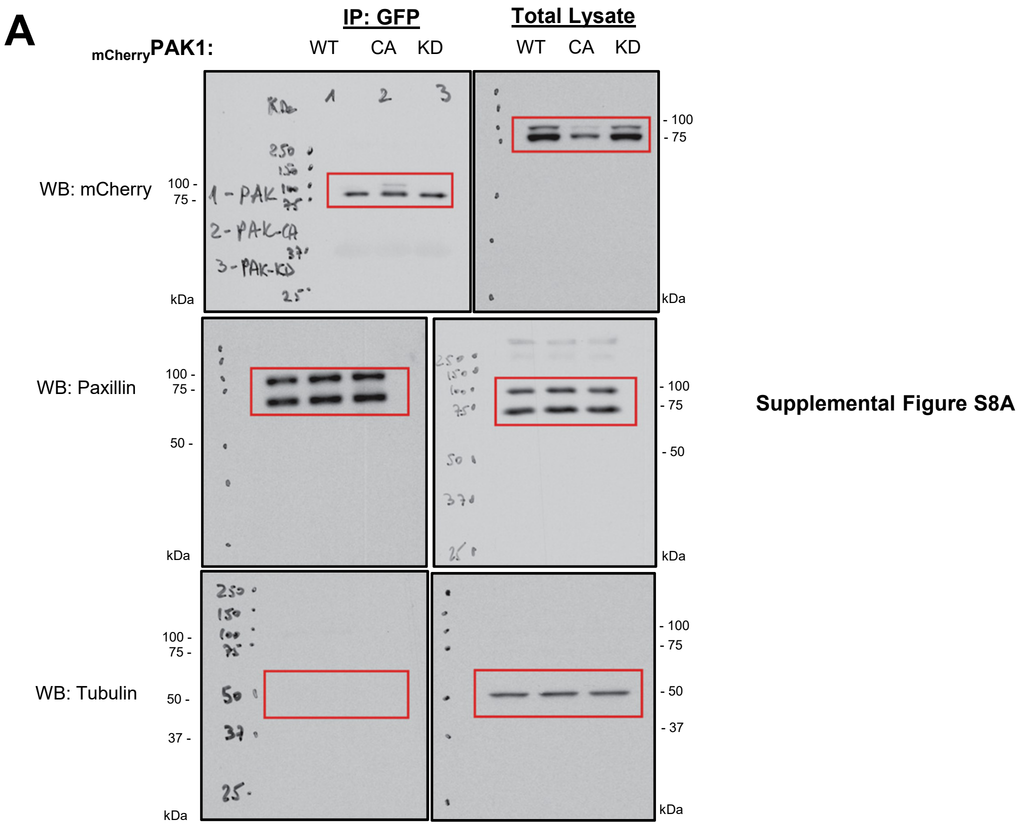

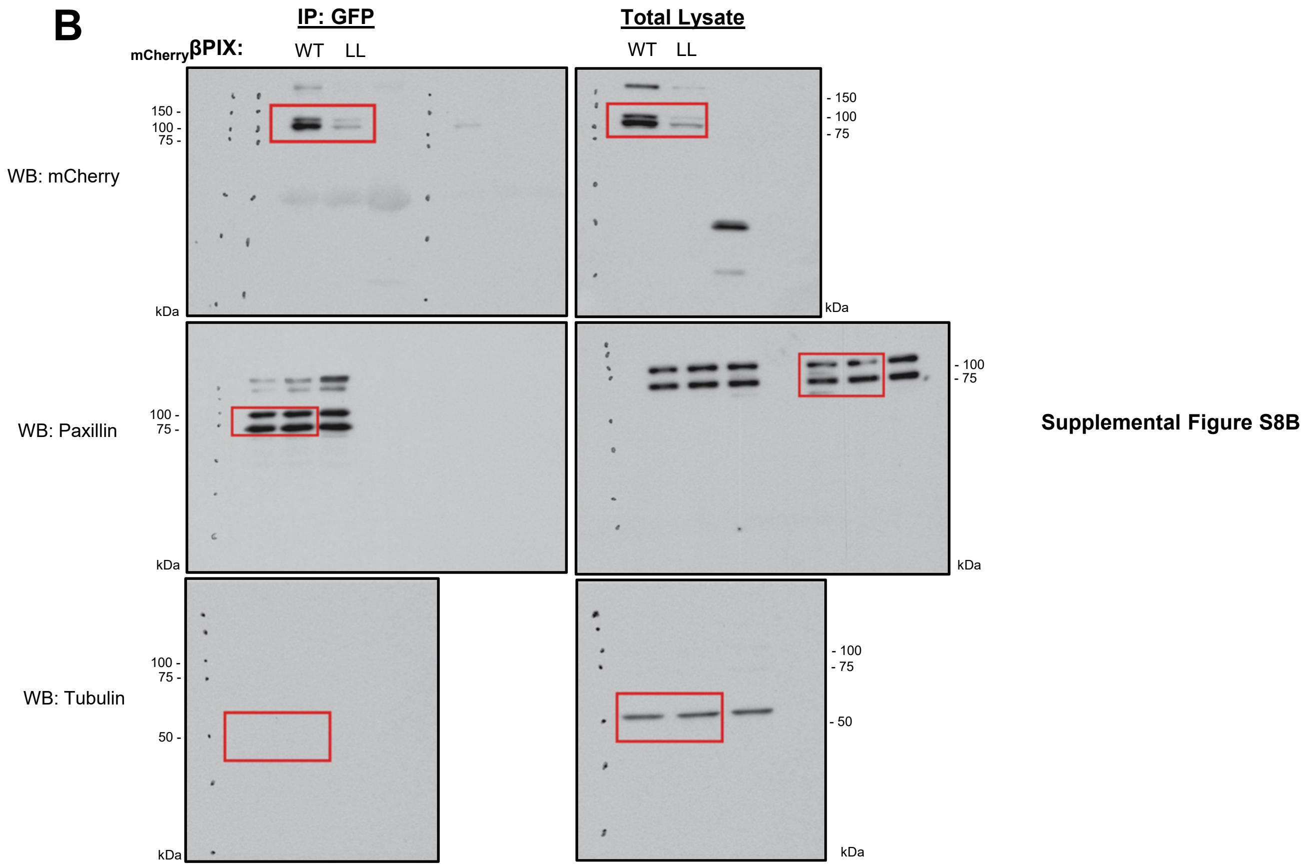

Rajah and Boudreau et al. Supplemental Figure S9

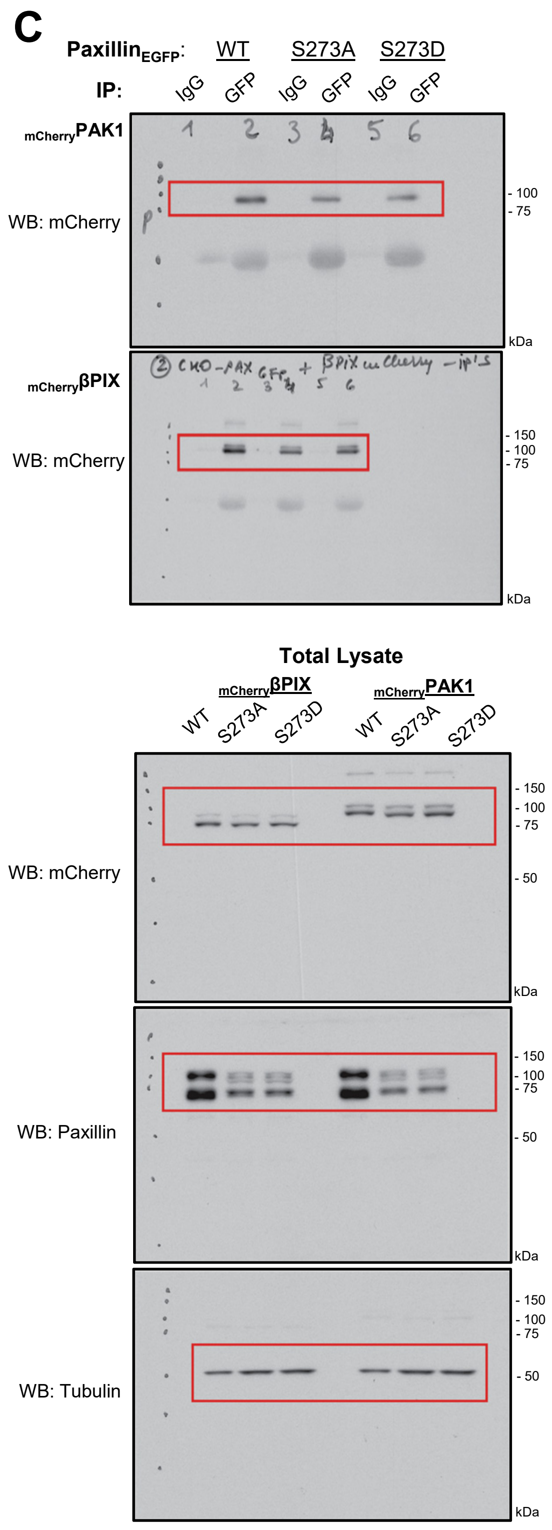

Supplemental Figure S8C

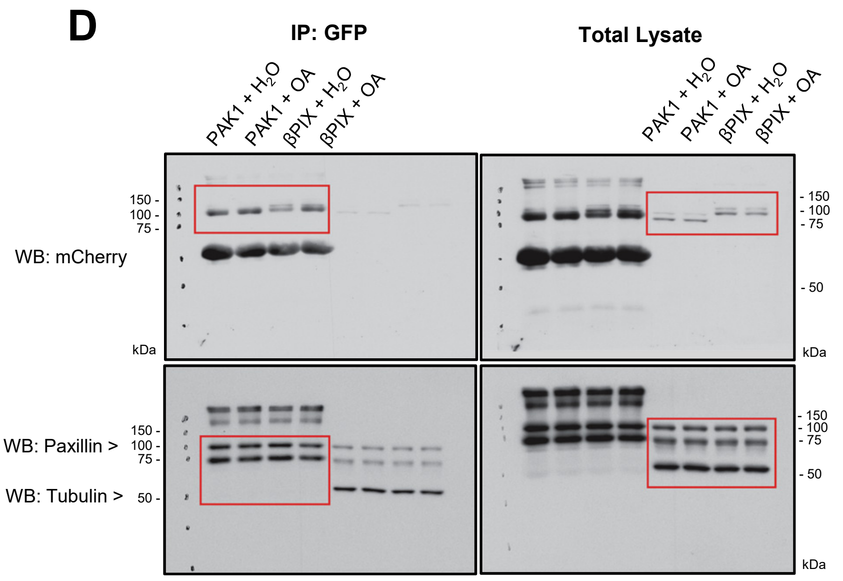

Supplemental Figure S8D

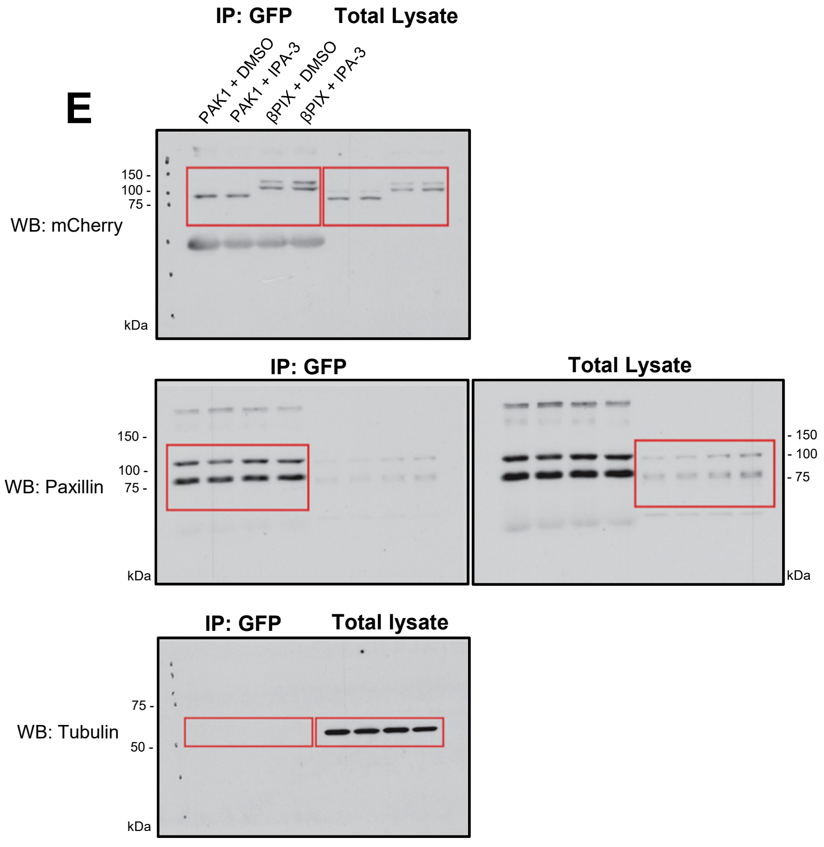

Supplemental Figure S8E
